# LncRNA TMEM99 Complexes with IGF2BP2 to Inhibit Autophagy in Lung Adenocarcinoma

**DOI:** 10.1002/advs.202507871

**Published:** 2025-07-24

**Authors:** Zhigang Wu, Yue Zhao, Yizhou Peng, Pengcheng Liu, Qixuan Huang, Yang Wo, Yunjian Pan, DongDong Zheng, Chongze Yuan, Yan Shang, Xiao Chen, Hui Hong, Yihua Sun

**Affiliations:** ^1^ Department of Thoracic Surgery Fudan University Shanghai Cancer Center Department of Oncology Shanghai Medical College Fudan University Shanghai 200032 China; ^2^ Department of Otolaryngology‐Head and Neck Surgery Shanghai Ninth People's Hospital Shanghai Jiaotong University School of Medicine Shanghai 200011 China; ^3^ Department of Thoracic Surgery I The Third Affiliated Hospital of Kunming Medical University (Yunnan Cancer Hospital Yunnan Cancer Center) Kunming 650000 China; ^4^ Department of Ultrasound Fudan University Shanghai Cancer Center Department of Oncology Shanghai Medical College Fudan University Shanghai 200032 China

**Keywords:** autophagy, FUBP3, Insulin‐like growth factor 2 mRNA‐binding protein 2 (IGF2BP2), lung adenocarcinoma, TMEM99

## Abstract

Lung adenocarcinoma (LUAD), the most common type of lung cancer, has a poor prognosis. Long noncoding RNAs (lncRNAs) play a key role in LUAD progression, yet the biological role of lncRNA TMEM99 remains unexplored. In this study, its function in inhibiting autophagy in LUAD is explored. Using RNA sequencing and quantitative reverse transcription PCR (qRT‐PCR), TMEM99 is found upregulated in LUAD tissues and cell lines, correlating with poor patient outcomes. In vivo and in vitro assays confirmed that TMEM99 promotes cell proliferation, migration, and invasion, and inhibits autophagy. Mechanistically, the 3′ end of TMEM99 binds to the K‑homology domain 1 (KH1) and KH4 domains of far upstream element‑binding protein 3 (FUBP3), stabilizing its protein. The TMEM99‐FUBP3 complex binds to p21 mRNA and recruits IGF2BP2 in an N⁶‑methyladenosine (m⁶A)‐dependent manner, which enhances mRNA stability and translation efficiency. This study reveals that TMEM99 plays a crucial regulatory role in LUAD autophagy and presents a novel cytoplasmic regulatory mechanism contributing to LUAD progression.

## Graphical abstract

LncRNA TMEM99 is significantly upregulated in lung adenocarcinoma (LUAD) and correlates with poor prognosis. It promotes LUAD cell proliferation, migration, and invasion while suppressing autophagy. Mechanistically, TMEM99 binds FUBP3 and recruits IGF2BP2 in an m⁶A‐dependent manner to stabilize and enhance p21 mRNA translation, uncovering a novel cytoplasmic regulatory mechanism driving LUAD progression.

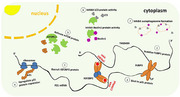

## 1 Introduction

Lung carcinoma remains the most prevalent and deadliest malignancy worldwide. In 2022, there were ≈2.5 million new cases and 1.8 million deaths attributed to lung cancer, accounting for 12.4% of all new cancer cases and 18.7% of all cancer‐related deaths globally.^[^ ^1^ ^]^ Adenocarcinoma, the predominant histological subtype of lung carcinoma, is increasingly prevalent, particularly among non‐smokers. In 2022, it represented 45.6% of lung cancer cases in men and 59.7% in women, with a significant portion of these cases linked to air pollution, especially in East Asia.^[^ ^2^ ^]^ Despite advancements in targeted therapies, such as pembrolizumab, sintilimab, and crizotinib, the prognosis for lung cancer patients remains poor. This underscores the necessity for continued research into the complex etiopathogenesis of lung carcinoma and the identification of novel therapeutic targets.^[^ ^3^, ^4^, ^5^ ^]^

Although myriad genes undergo transcription in the human genome, a mere 2% culminate in protein synthesis, engendering an extensive array of non‐coding RNAs.^[^ ^6^ ^]^ Long‐chain noncoding RNAs (lncRNAs) are designated for those exceeding 200 nucleotides in length.^[^ ^7^ ^]^ Historically, lncRNAs have been postulated to exert intricate and pivotal roles in oncogenesis, modulating comprehensive cellular epigenetic landscapes through interactions with DNA, mRNA, miRNA, and proteins.^[^ ^8^ ^]^ Yet, while numerous lncRNAs exhibit enhanced expression, their overall abundance remains modest in contrast to mRNAs. Many lncRNAs remain enigmatic due to their nuanced conservation patterns.^[^ ^9^ ^]^ The intricate functionalities and mechanisms of lncRNAs, including m^6^A modifications, remain largely enigmatic.

m^6^A modifications influence a wide range of biological processes, including pre‐mRNA splicing, nuclear export, mRNA stability, and translation. These modifications are increasingly implicated in oncogenesis.^[^ ^10^ ^]^ As elucidated in prior studies, canonical writer proteins encompass METTL3, METTL14, and WTAP,^[^ ^11^, ^12^, ^13^ ^]^ facilitating the methylation of specific adenine residues. Conversely, erasers like FTO and ALKBH5 demethylate these sites.^[^ ^14^, ^15^ ^]^ Predominantly, the functional implication of such methylation is discerned by reader proteins, notably the YTHDF and IGF2BPs families.^[^ ^16^, ^17^ ^]^ Contemporary findings underscore the significance of IGF2BP2, a salient m^6^A reader, stabilizing Gys2 mRNA by modulating downstream genes, thus augmenting hepatic glycogen synthesis and conserving hepatic glycogen reservoirs.^[^ ^18^ ^]^ The diverse roles of m^6^A, encompassing pre‐mRNA splicing, nucleic translocation, mRNA stabilization, and translational enhancement, have implicated oncogenesis.^[^ ^19^ ^]^ It's noteworthy that lncRNAs modulate each facet of m^6^A dynamics, associating with various m^6^A modifiers.^[^ ^20^, ^21^, ^22^ ^]^ Notwithstanding, in lung carcinoma, the lncRNA‐mediated regulation via the m^6^A axis remains underexplored, necessitating a comprehensive investigation.

In this study, we delineate the mechanisms of m^6^A‐associated lncRNAs in lung adenocarcinoma pathogenesis. Our findings uncover a novel lncRNAs, TMEM99, capable of stabilizing the FUBP3 protein and recruiting IGF2BP2 via m6A‐dependent recognition at the p21 mRNA locus, thereby enhancing its translation and inhibiting the occurrence of autophagy. This research elucidates novel avenues in lung carcinoma pathogenesis, fostering advancements in prognostic assessments and targeted therapeutic interventions.

## 2 Results

### 2.1 TMEM99 is a Key Oncogene in Lung Adenocarcinoma Development

**Figure 1:**
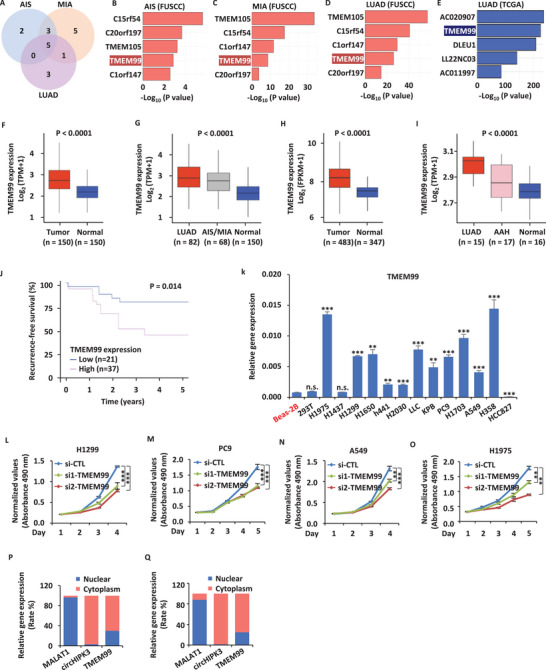
TMEM99 is a key oncogene in lung adenocarcinoma development. A) Significantly up‐regulated lncRNAs (FC > 1.5, *p* < 0.01) in AIS versus N, MIA versus N, and LUAD versus N are shown via a Venn diagram. B‐D) Overlapping lncRNAs from (A) in AIS (B), MIA (C), and LUAD (D). E) Top 5 enriched lncRNAs in LUAD from TCGA. F) TMEM99 expression in LUAD and adjacent normal tissues from FUSCC (*p* < 0.0001). G) TMEM99 expression in AIS/MIA, invasive adenocarcinoma, and normal lung tissues in FUSCC (*p* < 0.0001). H) TMEM99 expression in LUAD and normal tissues in TCGA‐LUAD cohort (*p* < 0.0001). I) TMEM99 expression in AAH, invasive adenocarcinoma, and normal lung tissues in Kadara's cohort (*p* < 0.0001). J) TMEM99 impact on Recurrence‐Free Survival (FRS) in LUAD from FUSCC (*p* = 0.014). K) TMEM99 expression in Beas‐2B, 293T, and LUAD cell lines by qRT‐PCR (± SEM; **p < 0.01; ***p < 0.001; n.s. not significant). L‐O) H1299 (L), PC9 (M), A549 (N), and H1975 (O) cells transfected with control or TMEM99‐specific siRNAs (si1‐TMEM99 and si2‐TMEM99) assessed by proliferation assays (± SEM; **p < 0.01; ***p < 0.001). P, Q) TMEM99 subcellular localization via nuclear‐cytoplasmic assays in H1299 (P) and PC9 (Q) cells, using MALAT1 as nuclear and circHIPK3 as cytoplasmic markers. The data are shown as the mean ± SD (*n* = 3). **p < 0.01, ***p < 0.001, by two‐tailed unpaired Student's t test (F‐I), one‐way ANOVA (K‐O) with Dunnett's post hoc test, and by the log‐rank test (J). AIS, Adenocarcinoma in situ; MIA, Minimally invasive adenocarcinoma; N, Normal tissue; AAH, Atypical adenomatous hyperplasia; FUSCC, Fudan University Shanghai Cancer Center.

We analyzed RNA sequencing (RNAseq) data from 150 lung adenocarcinoma samples and their matched normal lung tissues.^[^ ^23^, ^24^ ^]^ The LUAD samples were divided into adenocarcinoma in situ (AIS), minimally invasive adenocarcinoma (MIA), and LUAD based on pathological classification. Differentially expressed lncRNAs were identified for each pathological subgroup against normal lung tissues (**Figure** **1**A–D). Of the 10, 14, and 9 significantly up‐regulated lncRNAs for each comparison, 5 lncRNAs were consistently elevated in all three comparisons (Figure 1A): C15orf54, C20orf197, TMEM105, TMEM99, and C1orf147 (Figure 1B–D). Similar results were obtained from the RNAseq analysis of the The Cancer Genome Atlas (TCGA)‐LUAD cohort (Figure 1E). Notably, TMEM99 ranked prominently in both our sequencing data (FUSCC) and the TCGA‐LUAD cohort. Volcano plots also highlighted TMEM99's significant position (Figure S1A–D, Supporting Information). In Fudan University Shanghai Cancer Center (FUSCC), TMEM99 expression was significantly elevated in cancer and positively correlated with cancer progression (Figure 1F,G). RNAseq data from another cohort showed the same trend (Figure 1H), and TMEM99 was higher in LUAD than in atypical adenomatous hyperplasia (AAH) (Figure 1I).^[^ ^25^ ^]^ Among the invasive lung adenocarcinoma patients, 58 had follow‐up data available. Analysis revealed that cancer patients with different TMEM99 expression levels had significantly different relapse‐free survival (RFS) rates (*p* = 0.014), with higher TMEM99 expression correlating with worse survival outcomes (Figure 1J). Furthermore, univariate and multivariate Cox regression analyses confirmed that TMEM99 expression is an independent prognostic factor for RFS, with hazard ratios of 7.505 (95% CI: 1.751–32.175, *p* = 0.00665) and 7.738 (95% CI: 1.730–34.614, *p* = 0.00743), respectively. These results underscore its significant association with poorer survival outcomes. Although there wasn't a significant difference in our overall survival (OS) data, there was a trend suggesting that patients with higher TMEM99 expression had poorer survival outcomes (Figure S2, Supporting Information). Hence, we chose TMEM99 for further analysis. Based on expression profiles across different cell lines, TMEM99 expression was significantly elevated in most lung adenocarcinoma cell lines compared to the Beas‐2 B cell line (Figure 1K). We knocked down TMEM99 levels using siRNA (Figure S3A–D, Supporting Information) and found that TMEM99 significantly affects cell proliferation, especially in the H1299, PC9, A549, and H1975 cell lines (Figure 1L–O). Based on nucleocytoplasmic separation, we found that TMEM99 localized in both the nucleus and cytoplasm, but predominantly in the cytoplasm (Figure 1P,Q).

### 2.2 LncRNA TMEM99 Promotes Proliferation, Migration, and Invasion of H1299 and PC9 Cells

**Figure 2:**
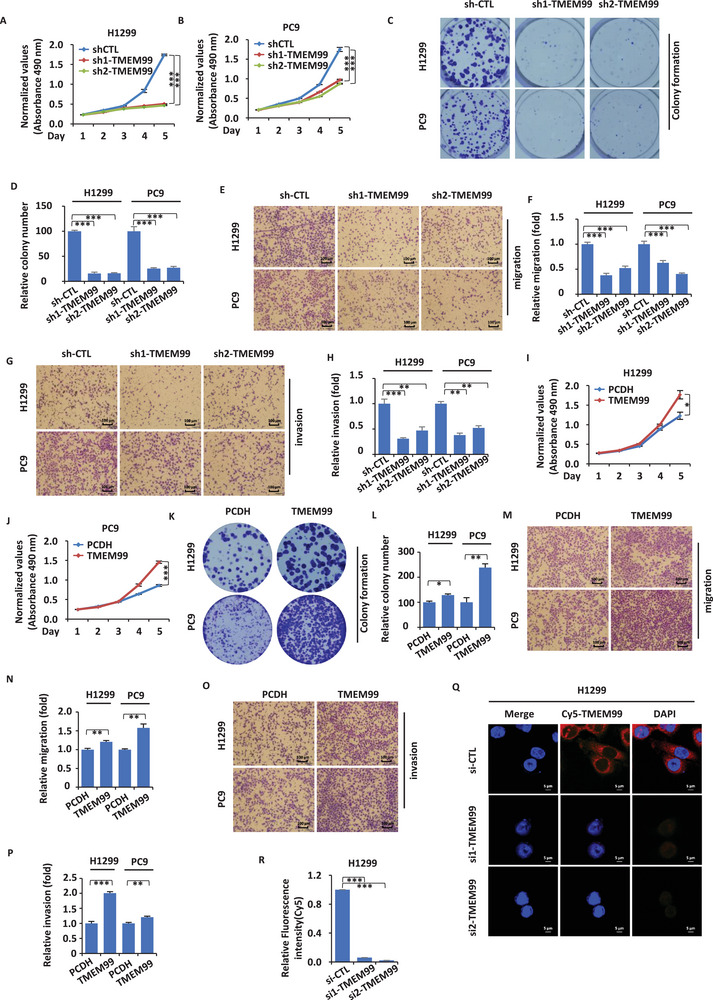
LncRNA TMEM99 promotes proliferation, migration, and invasion of H1299 and PC9 cells. A, B) H1299 (A) and PC9 (B) cells infected with control shRNA (sh‐CTL) or TMEM99‐specific shRNAs (sh1‐TMEM99, sh2‐TMEM99) were analyzed for proliferation (± SEM; ***p < 0.001). C) H1299 and PC9 cells, as described in (A‐B), were analyzed by colony formation assay. D) Quantification of colonies from (C) (± SEM; ***p < 0.001). E, G) H1299 and PC9 cells, as described in (A‐B), subjected to migration (E) and invasion (G) assays. F, H) Quantification of migrated (F) and invaded (H) cells from (E, G) (± SEM; **p < 0.01; ***p < 0.001). I, J) H1299 (I) and PC9 (J) cells transfected with control vector (PCDH) or TMEM99‐expressing vector, followed by proliferation assay (± SEM; *p < 0.05; ***p < 0.001). K) H1299 and PC9 cells, as described in (I‐J), were analyzed by colony formation assay. L) Quantification of colonies from (K) (± SEM; *p < 0.05; **p < 0.01). M, O) H1299 and PC9 cells, as described in (I‐J), subjected to migration (M) and invasion (O) assays. N, P) Quantification of migrated (N) and invaded (P) cells from (M, O) (± SEM; **p < 0.01; ***p < 0.001). Q) RNA‐FISH analysis in H1299 cells from (A) using TMEM99‐specific probe (Cy5‐TMEM99). Red: TMEM99; Blue: DAPI. R; Scale bar = 5 µm.) Quantification of fluorescence intensity (Cy5) (± SEM; ***p < 0.001). The data are shown as the mean ± SD (*n* = 3). **p < 0.01, ***p < 0.001, by two‐tailed unpaired Student's t test (I, J, L, N, and P) and one‐way ANOVA (A, B, D, F, H, and R) with Dunnett's post hoc test. shRNA, short hairpin RNA; sh‐CTL, control shRNA; sh1‐TMEM99/sh2‐TMEM99, TMEM99‐specific shRNA sequences 1 and 2; PCDH, control vector; RNA‐FISH, RNA fluorescence in situ hybridization; Cy5, cyanine 5, a fluorescent dye; DAPI, 4′,6‐diamidino‐2‐phenylindole, a nuclear stain.

We further conducted functional validation in two cell lines (H1299 and PC9), specifically constructing stable cell lines by generating stable cell lines with either TMEM99 knockdown or overexpression (Figure S3E–H, Supporting Information). Through cell counting kit‑8 (CCK8) assays, we observed that knocking down TMEM99 by shRNA exhibited a significant suppression in proliferation (**Figure** **2**A,B). Conversely, upon overexpression of TMEM99, a significant increase in proliferative capacity was observed (Figure 2I,J). Colony formation assays further revealed that TMEM99 promotes colony formation in cells (Figure 2C,D,K–L). Given the oncogenic role of TMEM99 in cell proliferation, we next examined its effect on cell motility. We employed transwell assays and found that knocking down or overexpressing TMEM99 reduced or enhanced cell migration and invasion, respectively (Figure 2E–H,M–P). Using FISH assays, we verified siRNA specificity and examined the subcellular localization of TMEM99. This provided direction for subsequent mechanism research. Verification showed that TMEM99 is primarily located in the cytoplasm (Figure 2Q,R).

### 2.3 LncRNA TMEM99 Interacts with FUBP3 Protein

**Figure 3:**
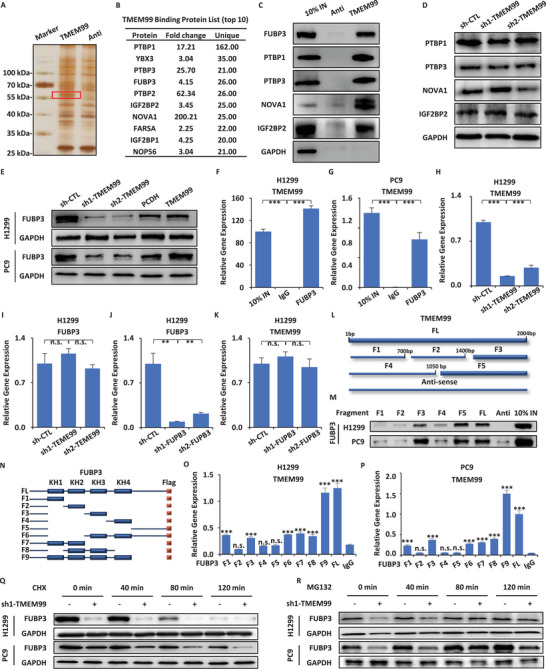
LncRNA TMEM99 interacts with FUBP3 protein. A) In vitro biotinylated TMEM99 RNA pull‐down followed by silver staining in H1299; antisense (Anti) used as a negative control. B) TMEM99‐associated proteins were purified and analyzed by mass spectrometry; the top 10 identified proteins are shown. C) TMEM99 RNA‐pull down assay with FUBP3, PTBP1, PTBP3, NOVA1, IGF2BP2 levels analyzed by IB in H1299; GAPDH as loading control; Anti and 10% input (10% IN) included. D) H1299 cells infected with control or TMEM99‐specific shRNAs to examine PTBP1, PTBP3, NOVA1, IGF2BP2 expression by IB; GAPDH as loading control. E) H1299 and PC9 cells infected with sh‐CTL, sh1/2‐TMEM99, PCDH, TMEM99‐expressing vector to examine FUBP3 expression by IB; GAPDH as control. F, G) FUBP3 RIP assay in H1299 (F) and PC9 (G) with TMEM99 expression measured by qRT‐PCR (± SEM; ***p < 0.001); 10% input as positive and IgG as negative controls. H, I) H1299 cells infected with sh‐CTL, sh1/2‐TMEM99 followed by qRT‐PCR to examine TMEM99 (H) and FUBP3 mRNA (I) expression (± SEM; ***p < 0.001; n.s. not significant). J, K) H1299 cells infected with sh‐CTL, sh1/2‐FUBP3 followed by qRT‐PCR to examine FUBP3 (J) and TMEM99 (K) expression (± SEM; **p < 0.01; n.s. not significant). L) TMEM99 isoforms shown: Full Length (FL), Fragments (F1‐F5), and Anti. M) In vitro biotinylated TMEM99 isoforms RNA‐pull down assay in H1299 or PC9 cells to examine FUBP3 by IB; 10% input included. N) Schematic of FUBP3 domain architecture and truncations (KH1‐KH4) with Flag tag, Full Length (FL), Fragments (F1‐F9). O, P) RIP assay with FUBP3 truncations and qRT‐PCR to examine TMEM99 enrichment in H1299 (O) or PC9 (P) cells (± SEM; ***p < 0.001; n.s. not significant). Q, R) H1299 or PC9 cells infected with sh‐CTL or sh1‐TMEM99 treated with CHX (Q) or MG132 (R), followed by IB using FUBP3 antibody; GAPDH as control. The data are shown as the mean ± SD (*n* = 3). *p < 0.05, **p < 0.01, ***p < 0.001, by two‐tailed unpaired Student's t test (F, G) and one‐way ANOVA followed by Dunnett's post hoc test (H‐K, O, and P). Anti, antisense RNA; IB, immunoblotting; RIP, RNA immunoprecipitation; sh1/2‐FUBP3, FUBP3‐specific shRNA sequences 1 and 2; CHX, cycloheximide, a protein synthesis inhibitor; KH1‐KH4, K homology domains 1–4 in FUBP3; FL, full‐length protein/RNA; F1‐F5/F1‐F9, fragments 1–5 or 1–9 of TMEM99 RNA or FUBP3 protein.

LncRNA‐protein interaction was known to be one of the important mechanisms. To investigate the functional mechanism of TMEM99, we aimed to identify the interacting proteins. We conducted an RNA pull‐down assay and observed distinct protein bands by silver staining (**Figure** **3**A). Using mass spectrometry, we shortlisted the top ten proteins based on their expression levels (Figure 3B). We selected proteins previously associated with proliferation for validation (FUBP3, PTBP1, PTBP3, NOVA1, IGF2BP2) using Immunoblotting (IB) and found our sequencing results to be consistent and reliable (Figure 3C; Figure S4, Supporting Information). To delve deeper into downstream proteins potentially affected by TMEM99, we knocked down TMEM99 and found that only FUBP3's expression level was suppressed (Figure 3D,E). Moreover, when TMEM99 was overexpressed, we detected a moderate increase in FUBP3 expression levels (Figure 3E), suggesting FUBP3 could be a downstream protein. To further validate the relationship between TMEM99 and FUBP3, we used a RNA immunoprecipitation (RIP) assay and confirmed that FUBP3 can effectively bind to TMEM99 (Figure 3F,G). However, after knocking down TMEM99, we discovered that the mRNA abundance of FUBP3 remained unchanged (Figure 3H,I). This indicates that TMEM99 regulates FUBP3 post‐transcriptionally, likely at the protein level. Similarly, reducing FUBP3 levels didn't affect TMEM99's expression (Figure 3J–K; Figure S5, Supporting Information). To pinpoint the binding region between TMEM99 and FUBP3, we divided TMEM99 into five truncated variants (Figure 3L) and found that FUBP3 showed strong binding affinity to F3 and F5 fragments of TMEM99 (Figure 3M). Based on FUBP3's structural domains, we created nine protein isoforms (Figure 3N). RIP assays revealed that TMEM99 primarily binds with FUBP3's KH1 and KH3 domains (Figure 3O,P). After treating cells with cycloheximide (CHX), we noticed a significant degradation in the control group's FUBP3 protein (Figure 3Q). Post MG132 treatment, protein accumulation was observed in cell lines, and the difference between sh‐CTL and sh1‐TMEM99 became increasingly minimal (Figure 3R). Our findings suggest that FUBP3 might be protected by TMEM99, delaying the protein's degradation.

### 2.4 FUBP3 Promotes Proliferation, Migration, and Invasion of H1299 and PC9 Cells

**Figure 4:**
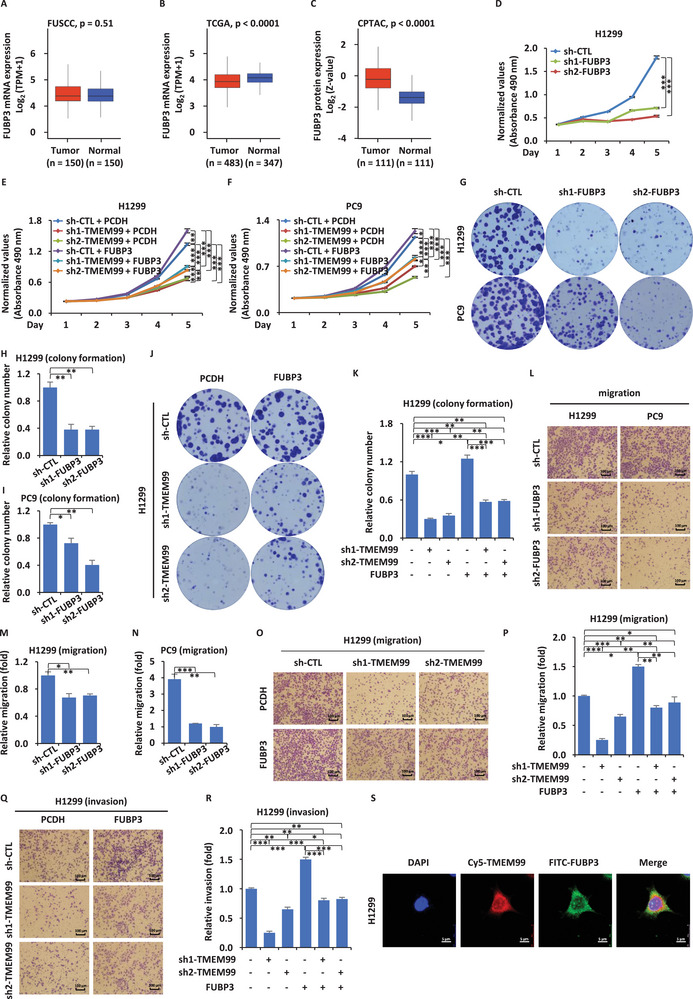
FUBP3 promotes proliferation, migration, and invasion of H1299 and PC9 cells. A‐C) FUBP3 expression in lung adenocarcinoma and adjacent normal tissues from FUSCC (*p* = 0.51) (A), TCGA (*p* < 0.0001) (B), and CPTAC (*p* < 0.0001) (C) databases. D) H1299 cells infected with sh‐CTL, sh1/2‐FUBP3 were analyzed for proliferation (± SEM; ***p < 0.001). E, F) H1299 (E) and PC9 (F) cells infected with sh‐CTL, sh1/2‐TMEM99, and transfected with or without FUBP3‐expressing vector, followed by proliferation assay (± SEM; *p < 0.05; **p < 0.01; ***p < 0.001). PCDH: control vector. G) H1299 and PC9 cells infected with sh‐CTL, sh1/2‐FUBP3 subjected to colony formation assay. H, I) Quantification of colony numbers from (G) (± SEM; *p < 0.05; **p < 0.01). J, K) H1299 cells from (E) subjected to colony formation assay (J), with colony quantification in (K) (± SEM; *p < 0.05; **p < 0.01; ***p < 0.001). L) H1299 and PC9 cells infected with sh‐CTL, sh1/2‐FUBP3 subjected to migration assay. Scale bar = 100 µm. M,N) Quantification of migrated cells from (L) (± SEM; *p < 0.05; **p < 0.01; ***p < 0.001). O, Q) H1299 cells from (E) subjected to migration (O) and invasion (Q) assays. Scale bar = 100 µm. P, R) Quantification of migrated and invaded cells from (O, Q) (± SEM; *p < 0.05; **p < 0.01; ***p < 0.001). S) H1299 cells transfected with TMEM99 or FUBP3‐expressing vectors analyzed by FISH‐immunofluorescence using probes for TMEM99 (Cy5‐TMEM99) and FUBP3 (FITC‐FUBP3). Blue: DAPI; Red: TMEM99; Green: FUBP3. Scale bar = 5 µm. The data are shown as the mean ± SD (*n* = 3). *p < 0.05, **p < 0.01, ***p < 0.001, by two‐tailed unpaired Student's t test (A–C) and one‐way ANOVA followed by Dunnett's post hoc test (D‐F, H, I, K, M, N, P, and R). CPTAC, Clinical Proteomic Tumor Analysis Consortium; FITC, fluorescein isothiocyanate, fluorescent dye.

Using our own database, we investigated the mRNA changes of FUBP3 and found that there was no significant difference between cancer tissues and adjacent normal tissues (**Figure** **4**A). Surprisingly, we observed that mRNA levels in cancer tissues were even lower than in normal tissues, a finding consistent with data from the TCGA‐LUAD dataset (Figure 4B). However, CPTAC database analysis showed a significant increase in FUBP3 protein levels in cancer tissues (Figure 4C).

To understand the functional role of FUBP3, we knocked it down and found through CCK8 assays that its depletion significantly inhibited cell proliferation (Figure 4D; Figure S6A, Supporting Information). We further carried out a rescue assay by overexpressing FUBP3 after knocking down TMEM99. We observed a partial rescue of cell proliferation upon FUBP3 overexpression (Figure 4E,F). The results from the clone formation assay were consistent with the CCK8 findings (Figure 4G–K). To assess the impact of FUBP3 on migration and invasion, we conducted transwell assays and found that FUBP3 could indeed promote these cellular functions (Figure 4L–N; Figure S6D,F, Supporting Information). Moreover, rescue assays validated that FUBP3 could counteract the negative effects brought about by TMEM99 knockdown (Figure 4O–R; Figure S6B,C,G,H, Supporting Information). Interestingly, we noted that in the rescue assays, these results suggest that while FUBP3 contributes to these phenotypes, other factors may also be involved. Using combined RNA FISH and immunofluorescence, we observed that TMEM99 and FUBP3 co‐localize in the cytoplasm (Figure 4S).

### 2.5 TMEM99 Regulates p21 via the FUBP3 Signaling Axis

**Figure 5:**
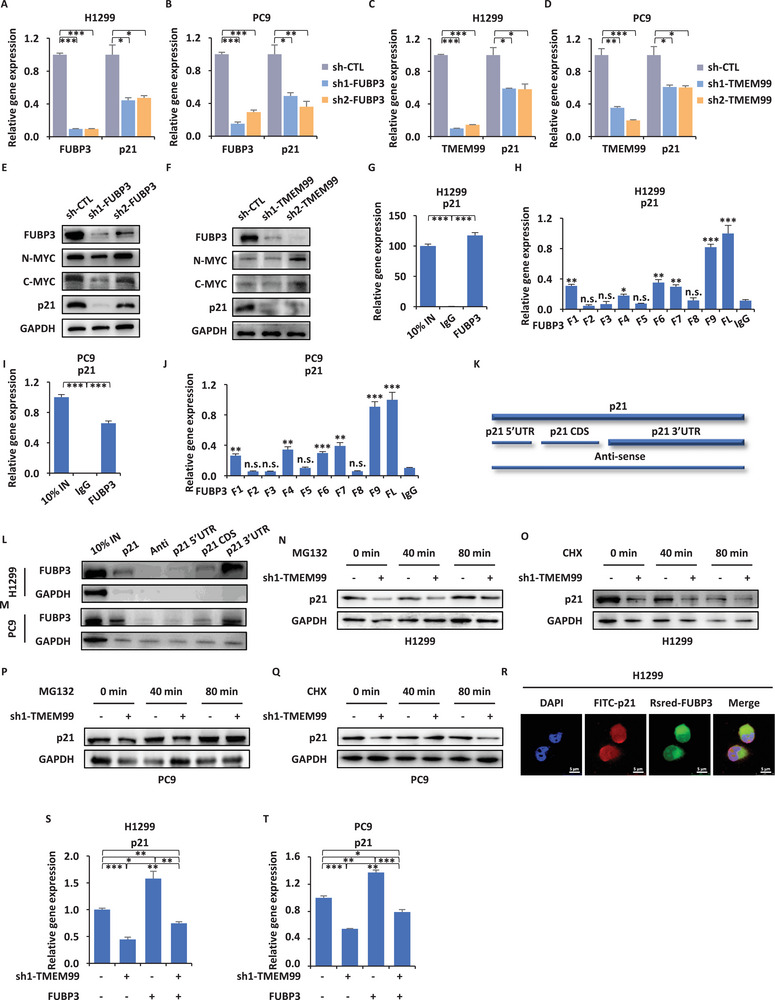
TMEM99 regulates p21 via the FUBP3 signaling axis. A, B) qRT‐PCR analysis of FUBP3 and p21 expression in H1299 (A) and PC9 (B) cells infected with sh‐CTL, sh1/2‐FUBP3 (± SEM; *p < 0.05; **p < 0.01; ***p < 0.001). C, D) qRT‐PCR analysis of TMEM99 and p21 expression in H1299 (C) and PC9 (D) cells infected with sh‐CTL, sh1/2‐TMEM99 (± SEM; *p < 0.05; ***p < 0.001). E, F) IB analysis of FUBP3, N‐MYC, C‐MYC, and p21 protein levels in H1299 cells infected with sh‐CTL, sh1/2‐FUBP3 (E) or sh1/2‐TMEM99 (F); GAPDH as control. G, I) FUBP3 RIP assay in H1299 (G) and PC9 (I) cells with p21 expression measured by qRT‐PCR (± SEM; ***p < 0.001); 10% input as positive, IgG as negative controls. H, J) In vitro FUBP3 truncation RIP assay and qRT‐PCR analysis to examine p21 enrichment in H1299 (H) and PC9 (J) cells (± SEM; *p < 0.05; **p < 0.01; ***p < 0.001; n.s. not significant). K) p21 isoforms: p21, p21 5′UTR, p21 CDS, p21 3′UTR, and Anti. L, M) RNA‐pull down assay with biotinylated p21 isoforms in H1299 (L) and PC9 (M) cells to examine FUBP3 protein level by IB; 10% input included. N‐Q) H1299 (N, O) and PC9 (P, Q) cells infected with sh‐CTL or sh1‐TMEM99 treated with MG132 (N, P) or CHX (O, Q), followed by IB analysis using p21 antibody; GAPDH as control. R) FISH‐immunofluorescence analysis of H1299 cells transfected with p21 or FUBP3‐expressing vectors using p21 (FITC‐p21) and FUBP3 (RSred‐FUBP3) probes. Blue: DAPI; Red: p21; Green: FUBP3. Scale bar = 5 µm. S, T) qRT‐PCR analysis of p21 expression in H1299 (S) and PC9 (T) cells infected with sh‐CTL or sh1‐TMEM99, transfected with or without FUBP3‐expressing vector (± SEM; *p < 0.05; **p < 0.01; ***p < 0.001); PCDH as control vector. The data are shown as the mean ± SD (*n* = 3). *p < 0.05, **p < 0.01, ***p < 0.001, by two‐tailed unpaired Student's t test (I, G) and one‐way ANOVA followed by Dunnett's post hoc test (A‐D, H, J, S, and T). UTR, untranslated region; CDS, coding sequence; RSred, red fluorescent dye.

FUBP3 was reported to mainly function by binding to RNA and DNA,^[^ ^26^, ^27^ ^]^ but lacks the capacity to directly regulate transcription. We sought to understand how it influences downstream targets. Given that FUBP3 lacks protein interaction domains (https://www.uniprot.org/uniprotkb?query=FUBP3), we performed RNAseq analysis after knocking down FUBP3. GO and KEGG analyses showed that the cell cycle was mainly affected (Figure S7A,B, Supporting Information). Following the knockdown of either FUBP3 or TMEM99 resulted in a marked reduction in p21 mRNA expression (**Figure** **5**A–D). We individually knocked down FUBP3 or TMEM99 and detected the expression levels of FUBP3, N‐MYC, C‐MYC, p21, and GAPDH. The results revealed that only p21 expression was significantly down‐regulated, while the levels of N‐MYC and C‐MYC exhibited no significant changes (Figure 5E,F).

We further investigated the relationship between p21 and FUBP3. RIP assays confirmed direct binding of FUBP3 to p21 mRNA (Figure 5G,I). This binding mainly occurs at the KH1 and KH4 domains of FUBP3 (Figure 3N and Figure 5H,J). Using RNA pull‐down assays, we identified that FUBP3 predominantly binds to the 3′UTR region of p21 (Figure 5K–M). Through MG132 and CHX assays, suggesting that the regulatory effects primarily occur at the mRNA level, rather than through protein degradation (Figure 5N–Q). Similarly, we observed that p21 mRNA and FUBP3 co‐localize in the cytoplasm (Figure 5R).

Subsequently, through TMEM99 knocking down and FUBP3 rescue assay, we found that p21's restoration was not prominent (Figure 5S,T). This suggests that FUBP3 might not be the only factor affecting p21 expression.

### 2.6 IGF2BP2 is Involved in the TMEM99‐FUBP3‐p21 Regulatory Axis to Affect Cell Autophagy

**Figure 6:**
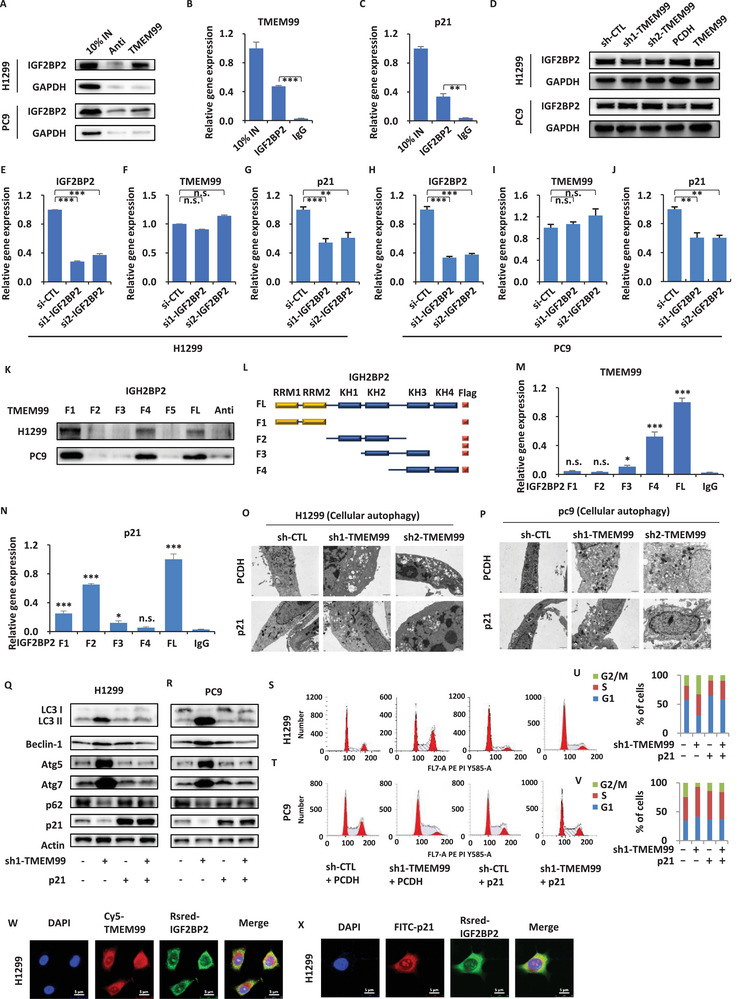
IGF2BP2 is involved in the TMEM99‐FUBP3‐p21 regulatory axis to affect cell autophagy. A) TMEM99 RNA‐pull down assay in H1299 and PC9 cells with IGF2BP2 protein levels analyzed by IB; GAPDH as loading control; 10% input included. B, C) IGF2BP2 RIP assay in H1299 cells with TMEM99 (B) and p21 (C) levels measured by qRT‐PCR (± SEM; **p < 0.01; ***p < 0.001); 10% input as positive, IgG as negative controls. D) H1299 and PC9 cells infected with sh‐CTL, sh1/2‐TMEM99, PCDH, or TMEM99‐expressing vector to examine IGF2BP2 expression by IB; GAPDH as control. E‐J) H1299 (E‐G) and PC9 (H‐J) cells infected with control or IGF2BP2‐specific siRNAs (si1/2‐IGF2BP2) were analyzed for IGF2BP2 (E, H), TMEM99 (F, I), and p21 (G, J) expression by qRT‐PCR (± SEM; **p < 0.01; ***p < 0.001; n.s. not significant). K) RNA‐pull down assay with TMEM99 isoforms in H1299 and PC9 cells to examine IGF2BP2 by IB. L) Schematic of IGF2BP2 domain architecture and truncations (RRM1, RRM2, KH1‐KH4) with Flag tag. M, N) RIP assay with IGF2BP2 truncations and qRT‐PCR to examine TMEM99 (M) and p21 (N) enrichment in H1299 cells (± SEM; *p < 0.05; ***p < 0.001; n.s. not significant). O, P) H1299 (O) and PC9 (P) cells infected with sh‐CTL, sh1/2‐TMEM99, transfected with or without p21‐expressing vector, were analyzed by electron microscopy; PCDH as a control vector. Scale bar = 1 µm. Q, R) Western blot analysis of H1299 (Q) and PC9 (R) cells transfected with sh‐CTL or sh1‐TMEM99, with or without p21 vector, examining Actin, beclin1, LC3, Atg5, ATG7, p62, and p21 expression; PCDH as control. S, T) Flow cytometry analysis of H1299 (Q) and PC9 (R) cells transfected with sh‐CTL or sh1‐TMEM99, with or without p21 vector, examining cell cycle. U, V) Quantification of cell cycle (G1, S, G2/M) from (S, T). W, X) FISH‐immunofluorescence analysis of H1299 (S) and PC9 (T) cells using probes for TMEM99 (Cy5‐TMEM99), IGF2BP2 (IGF2BP2‐RSred), and p21 (FITC‐p21). Blue: DAPI; Red: TMEM99; Green: IGF2BP2. Scale bar = 5 µm. The data are shown as the mean ± SD (*n* = 3). *p < 0.05, **p < 0.01, ***p < 0.001, by two‐tailed unpaired Student's t test (B, C) and one‐way ANOVA followed by Dunnett's post hoc test (E‐G, M, N). siRNA, small interfering RNA; si1/2‐IGF2BP2, IGF2BP2‐specific siRNA sequences 1 and 2; RRM1/RRM2, RNA recognition motif domains 1 and 2 in IGF2BP2; KH1‐KH4, K homology domains 1–4 in IGF2BP2; Flag tag, a peptide tag for protein detection; LC3, microtubule‐associated protein 1A/1B‐light chain 3, autophagy marker; Atg5, autophagy‐related 5; ATG7, autophagy‐related 7; p62, sequestosome‐1, autophagy substrate.

From our mass spectrometry results, we identified the presence of IGF2BP2, a protein previously reported as an m6A recognition protein. Intriguingly, previous studies have shown that p21 is subject to regulation via m6A methylation.^[^ ^28^, ^29^ ^]^ Therefore, we speculated that IGF2BP2 might be involved in the regulation of p21. As mentioned previously, we verified its interaction with TMEM99 using RNA pull‐down and observed that its binding was unaffected by the expression levels of TMEM99 (Figure 3C,D and **Figure** **6**A). Further, RIP assays confirmed its binding to TMEM99 and p21 (Figure 6B,C). However, after knocking down or overexpressing TMEM99, the expression levels of IGF2BP2 showed no significant changes (Figure 6D). Interestingly, upon knocking down IGF2BP2, TMEM99 expression remained unaffected, but p21 expression was significantly downregulated (Figure 6E–J). Subsequently, using RNA pull‐down, we found that IGF2BP2 binds to the N‐terminal region of TMEM99 (Figure 3L and Figure 6K). Through RIP assays targeting IGF2BP2, we observed that TMEM99 mainly binds to its KH3/KH4 domains, while p21 primarily binds to its KH1/KH2 domains (Figure 6L–N).

In previous reports, p21 has been frequently described as an oncogene involved in autophagy. We confirmed this via electron microscopy. Following the knockdown of TMEM99, a significant increase in autophagy was observed (Figure 6O,P). Upon rescue of p21 expression, autophagy was notably inhibited. Through Western blotting, we detected concurrent changes in proteins associated with autophagy. After knocking down TMEM99, we observed a significant upregulation of Beclin1, LC3‐II, Atg5, and Atg7, while the expression of p62 was notably reduced. Reintroduction of p21 reversed the expression patterns of key autophagy‐related proteins (Figure 6Q,R). Interestingly, following the same treatment, we discovered that p21 knockdown induced cell cycle arrest at G2/M and S phases in H1299, and at S phase in PC9 cells (Figure 6S–V). Immunofluorescence assays confirmed that IGF2BP2 co‐localizes with TMEM99 and p21 in the cytoplasm (Figure 6W,X).

### 2.7 TMEM99 Enhances p21 mRNA Stability by Interacting with FUBP3 and IGF2BP2

**Figure 7:**
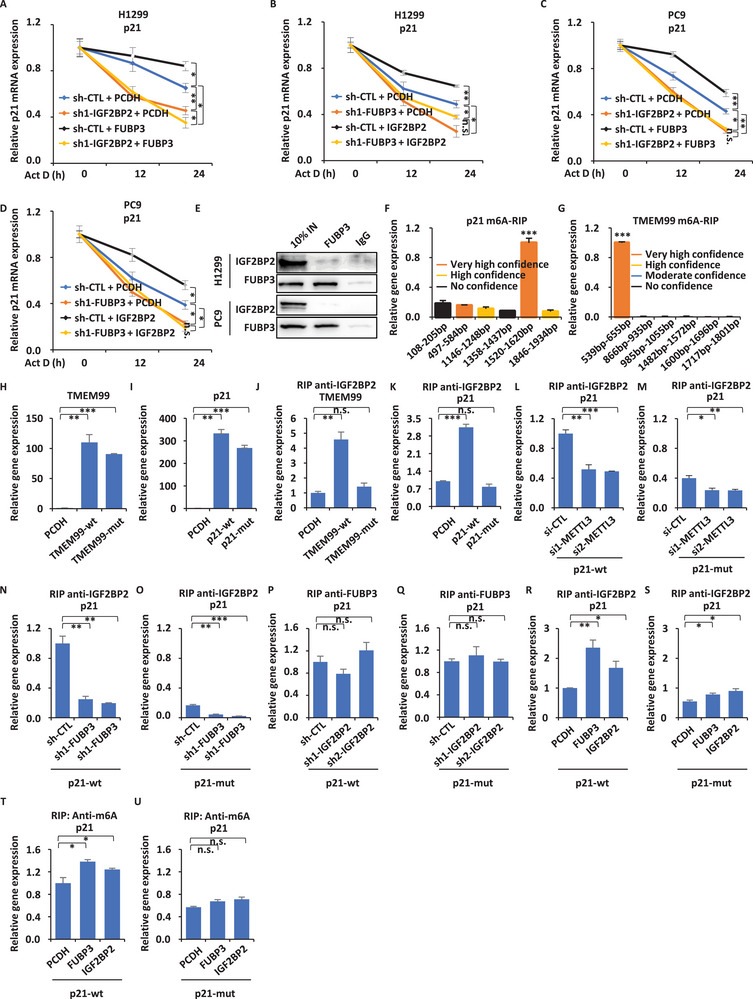
TMEM99 enhances p21 mRNA stability by interacting with FUBP3 and IGF2BP2. A, C) H1299 (A) and PC9 (C) cells infected with sh‐CTL or sh1‐IGF2BP2, transfected with control vector (PCDH) or FUBP3 vector, treated with Actinomycin D (Act D), followed by qRT‐PCR for p21 expression (± SEM; *p < 0.05; **p < 0.01). B, D) H1299 (B) and PC9 (D) cells infected with sh‐CTL or sh1‐FUBP3, transfected with PCDH or FUBP3 vector, treated with Act D, followed by qRT‐PCR for p21 expression (± SEM; *p < 0.05; **p < 0.01; n.s. not significant). E) Immunoprecipitation and IB of H1299 and PC9 cells transfected with IGF2BP2 or FUBP3 vectors; 10% input included. F, G) Mapping of m6A‐modified regions in TMEM99 and p21 mRNA via m6A RIP. H) qRT‐PCR of TMEM99 expression in H1299 cells transfected with PCDH, TMEM99‐wt, or TMEM99‐mut vectors (± SEM; **p < 0.01; ***p < 0.001). I) qRT‐PCR of p21 expression in H1299 cells transfected with PCDH, p21‐wt, or p21‐mut vectors (± SEM; **p < 0.01; ***p < 0.001). J, K) IGF2BP2 RIP in H1299 cells from (H, I) with TMEM99 (J) or p21 (K) enrichment measured by qRT‐PCR (± SEM; **p < 0.01; n.s. not significant). L, M) IGF2BP2 RIP in H1299 cells infected with si‐CTL or si1/2‐METTL3, transfected with p21‐wt (L) or p21‐mut (M), followed by qRT‐PCR for p21 enrichment (± SEM; *p < 0.05; **p < 0.01; ***p < 0.001). N, O) IGF2BP2 RIP in H1299 cells infected with sh‐CTL, sh1/2‐FUBP3, transfected with p21‐wt (N) or p21‐mut (O), followed by qRT‐PCR for p21 expression (± SEM; **p < 0.01; ***p < 0.001). P, Q) FUBP3 RIP in H1299 cells infected with sh‐CTL, sh1/2‐IGF2BP2, transfected with p21‐wt (P) or p21‐mut (Q), followed by qRT‐PCR for p21 expression (± SEM; n.s. not significant). R, S) IGF2BP2 RIP in H1299 cells transfected with PCDH, FUBP3, or IGF2BP2 vectors, followed by qRT‐PCR for p21‐wt (R) or p21‐mut (S) expression (± SEM; *p < 0.05; **p < 0.01). T, U) m6A RIP in H1299 cells from (R, S), followed by qRT‐PCR for p21 m6A site expression (± SEM; *p < 0.05; **p < 0.01). The data are shown as the mean ± SD (*n* = 3). *p < 0.05, **p < 0.01, ***p < 0.001, by one‐way ANOVA followed by Dunnett's post hoc test. Act D, actinomycin D, a transcriptional inhibitor; m6A, N6‐methyladenosine, an RNA methylation modification; wt, wild‐type; mut, mutant; METTL3, methyltransferase‐like 3, an m6A methyltransferase enzyme; m6A RIP, m6A‐specific RNA immunoprecipitation.

To further investigate the combined effects of IGF2BP2 and FUBP3 on p21, we alternately knocked down and overexpressed IGF2BP2 and FUBP3. After treating the cells with Actinomycin D, we found that knocking down either IGF2BP2 or FUBP3 could accelerate the degradation of p21 mRNA. Conversely, their overexpression inhibited this degradation. However, when one was knocked down and the other overexpressed simultaneously, this could not effectively rescue the expression of p21 (**Figure** **7**A–D), consistent with our previous findings. Seeking a deeper understanding of how FUBP3 and IGF2BP2 might work together to regulate p21, we conducted co‐immunoprecipitation (CoIP) assays using FUBP3. Co‐immunoprecipitation assays indicated that IGF2BP2 does not physically interact with FUBP3, suggesting that their effects on p21 are functionally coordinated rather than direct (Figure 7E).

Further probing into the mechanism of p21 action, we predicted m6A modification sites for TMEM99 and p21 using an online database (http://www.cuilab.cn/). Four potential sites were identified for TMEM99 (Figure S9A, Supporting Information) and four sites with high m6A modification probability for p21 (Figure S9B, Supporting Information). m6A RIP assays revealed that the predominant methylation sites were located at 1520–1620 bp in p21 and 539–655 bp in TMEM99 (Figure 7F,G). Using this data, we constructed m6A‐mutated plasmids for TMEM99 and p21 (Figure S9C,D, Supporting Information) and developed corresponding overexpression cell lines (Figure 7H,I). IGF2BP2 m6A RIP assays revealed a significant reduction in its binding capacity to both TMEM99 and p21 upon their m6A site mutation (Figure 7J,K). Previous reports suggested that p21 methylation is written by METTL3.^[^ ^28^, ^30^ ^]^ m6A RIP assays post METTL3 knockdown showed a noticeable reduction in p21 methylation (Figure 7L,M).

We further studied the regulatory mechanism of FUBP3 and IGF2BP2 on p21. After knocking down FUBP3, IGF2BP2's binding capacity to p21 was significantly inhibited, with severely diminished IGF2BP2 binding to p21 in the methylation‐mutant group, even under control (sh‐CTL) conditions (Figure 7N,O). We then investigated p21's binding to FUBP3 and found that knocking down IGF2BP2 or mutating p21's methylation sites had no effect on the binding capacity of FUBP3 to p21 (Figure 7P,Q). Through IGF2BP2 m6A RIP, we observed that in the p21‐wt and p21‐mut group, interestingly, co‐overexpression of FUBP3 and IGF2BP2 enhanced IGF2BP2's m6A‐dependent association with wild‐type p21, but not with the methylation‐deficient mutant (Figure 7R,S). Finally, upon overexpressing FUBP3 and IGF2BP2 and examining m6A RIP, we observed that while the wild‐type p21 group had a slight upregulation of p21, the p21 in the p21mut group remained largely unchanged (Figure 7T,U).

### 2.8 TMEM99 ASO Combined with PTX Synergistically Inhibited the Occurrence and Development of Tumors In Vivo

**Figure 8:**
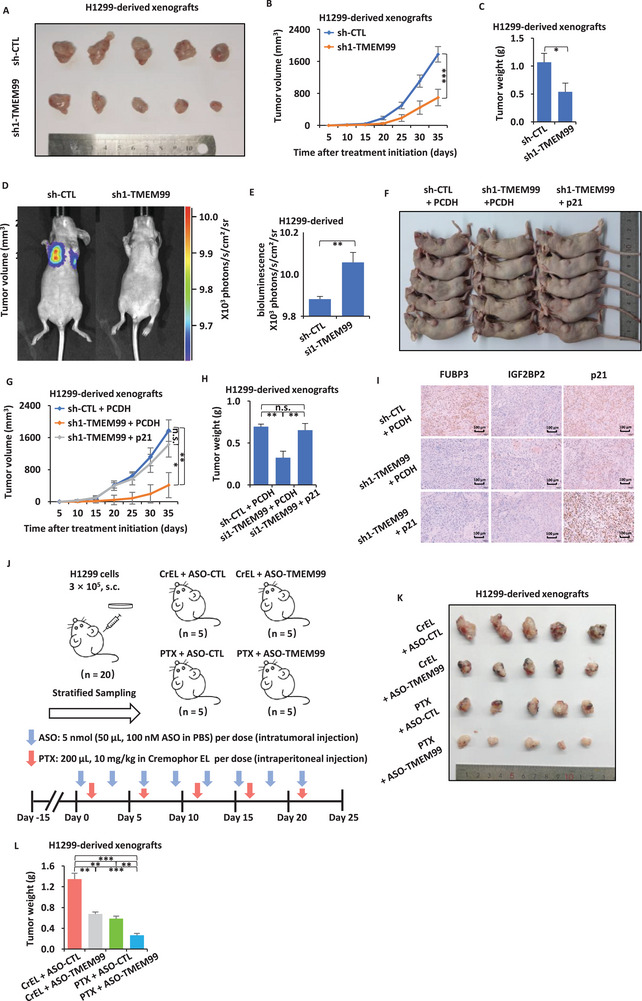
TMEM99 ASO combined with PTX synergistically inhibits tumor growth in *vivo*. A) H1299 cells infected with sh‐CTL or sh1‐TMEM99 were subcutaneously injected into BALB/C nude mice for xenograft assays; tumors shown. B) Tumor growth curve from (A) (± SEM; ***p < 0.001). C) Tumor weight from (A) (± SEM; *p < 0.05). D) Bioluminescence imaging of nude mice intravenously injected with H1299 cells expressing luciferase and transduced with sh‐CTL or sh1‐TMEM99. Imaging was performed using the IVIS system at week 3 post‐injection. Representative images are shown. E) Quantification of in vivo bioluminescence signal from mice injected with sh‐CTL or sh1‐TMEM99 H1299 cells (*n* = 5 per group) (± SEM; ***p < 0.01). F) H1299 cells infected with sh‐CTL or sh1‐TMEM99, transfected with PCDH or p21 vector, were subcutaneously injected into BALB/C nude mice for xenograft assays; tumors shown. G) Tumor growth curve from (E) (± SEM; *p < 0.05; **p < 0.01; n.s. not significant). H) Tumor weight from (E) (± SEM; **p < 0.01; n.s. not significant). I) Representative immunohistochemistry micrographs of FUBP3, IGF2BP2, and p21 in tumor tissues from (E). Scale bar = 100 µm. J‐L) BALB/c nude mice (*n* = 20) inoculated with H1299 cells, randomized, and treated with ASO‐TMEM99 (5 nmol, every 3 days) or PTX (10 mg kg^−1^, every 5 days) alone or combined. Treatment design shown (H); tumors excised, photographed (I), and weighed (J) (± SEM; **p < 0.01; ***p < 0.001). The data are shown as the mean ± SD (*n* = 5). *p < 0.05, **p < 0.01, ***p < 0.001, by two‐tailed unpaired Student's t test (B, C, E) and one‐way ANOVA followed by Dunnett's post hoc test (G, H, L). ASO, antisense oligonucleotide; PTX, paclitaxel, a chemotherapy drug; BALB/C nude mice, an immunodeficient mouse strain lacking T cells; IVIS, In Vivo Imaging System, for bioluminescence detection.

In this study, we carried out xenograft tumor assays using the H1299 cell line. TMEM99 knockdown significantly inhibited tumor growth in vivo (**Figure** **8**A–C). However, Overexpression of p21 partially restored tumor growth in the TMEM99‐deficient xenograft model (Figure 8F–H). Bioluminescence imaging revealed enhanced metastatic signals in TMEM99‐overexpressing tumors (Figure 8D,E). Immunohistochemistry showed that knocking down TMEM99 significantly reduced the expression of downstream proteins FUBP3 and p21, while IGF2BP2 levels remained unchanged, consistent with prior studies. As expected, p21 rescue restored p21 levels but had no impact on FUBP3 expression, further confirming the upstream position of TMEM99‐FUBP3 in the regulatory cascade (Figure 8I).

Historical studies have mentioned that the frontline chemotherapy drugs currently used clinically can effectively inhibit tumor growth. Paclitaxel is one of the most crucial drugs used in combined chemotherapy for lung cancer. However, its limited efficacy and the emergence of drug resistance mean that patient prognosis remains suboptimal. Previous reports showed that antisense oligonucleotides (ASO) can directly enter tissue cells and knock down intracellular lncRNAs. We hypothesized that combining ASO treatment with paclitaxel would enhance therapeutic efficacy in vivo. To test this, we treated mice with different drug regimens (Figure 8J). The results suggested that both the paclitaxel group and the ASO group effectively inhibited tumor growth. Moreover, the group treated with a combination of chemotherapy and ASO exhibited an even more pronounced inhibition of tumor growth (Figure 8K,L).

## 3 Discussion

LncRNAs participate in the occurrence and development of cancer through various mechanisms and affect the therapeutic effects of chemotherapy, targeted therapy, and even radiotherapy.^[^ ^31^, ^32^ ^]^ Through RNAseq, we aim to identify lncRNAs that play a crucial role in lung adenocarcinoma. We divided tissue samples into in situ carcinoma, microinvasive adenocarcinoma, adenocarcinoma, and their respective normal tissues. We observed a significant upregulation of TMEM99 expression across various pathological stages of cancer tissues. Similarly, through the TCGA database, TMEM99 was observed to be significantly upregulated in lung adenocarcinoma. Further analysis revealed that the expression of TMEM99 gradually increased with the progression of lung adenocarcinoma. Through survival analysis, we also identified that high expression of TMEM99 indicated poorer patient prognosis. In summary, TMEM99 may play a key role in the occurrence and development of LUAD, and it may be a new prognostic marker for lung adenocarcinoma. Consequently, we further carried out functional verification and noted that TMEM99 was elevated in almost all lung adenocarcinoma cell lines. Through the assays, TMEM99, as an oncogene, significantly affects cell proliferation, migration, and invasion. Furthermore, we found that it mainly exists in the cytoplasm, so we believe that TMEM99 may mainly play its oncogenic role in the cytoplasm.

LncRNAs can bind to proteins, RNA, and DNA, controlling gene expression levels, mRNA degradation, and translation regulation through mechanisms such as genomic imprinting, chromatin remodeling, cell cycle control, and splicing regulation.^[^ ^33^, ^34^ ^]^ To explore the interaction between lncRNAs and proteins in the cytoplasm, we performed protein spectrum analysis and expression verification of proteins interacting with TMEM99 through RNA pull‐down, and found that FUBP3 protein is regulated by TMEM99. Through segmented RNA pull‐down and RIP detection, we found that TMEM99 interacts with the KH1 and KH3 domains of FUBP3 through its tail sequence and stabilizes the structure of FUBP3 by binding, inhibiting the degradation process of FUBP3. Through sequencing data and database data analysis, we can see no significant difference in FUBP3 mRNA between lung adenocarcinoma cancer tissue and normal tissue. However, in the CPTAC database, FUBP3 protein expression is significantly elevated in lung adenocarcinoma tissues. This might be attributed to TMEM99 stabilizing the protein level of FUBP3 in the tissue, thereby allowing higher protein expression even when mRNA expression levels are low. Through assays, we found that FUBP3 has an oncogenic role in lung adenocarcinoma and promotes cell proliferation, migration, and invasion. Furthermore, after knocking down TMEM99 and rescuing FUBP3 expression, the cellular phenotype was partially restored with FUBP3 overexpression as expected. However, despite the abundance of FUBP3 protein far exceeding the control group level after the rescue assay, the cell phenotype could not be fully recovered. This suggests that FUBP3 might not be the only influencing factor.

In previous reports, FUBP3, mainly acting as a DNA and RNA binding protein, was found to be mainly composed of four KH subunits, all of which are RNA/DNA binding subunits. Gao et al.^[^ ^35^ ^]^ found that lncCMPK2 could bind with FUBP3, guiding FUBP3 to the upstream of the c‐Myc gene, affecting its transcription. Considering FUBP3's characteristic, we explored the changes in downstream RNA. Through RNAseq and verification analysis, we found that after knocking down FUBP3, p21 was most significantly down‐regulated in the downstream pathway. Moreover, after knocking down TMEM99, the mRNA and protein levels of p21 were also significantly down‐regulated, suggesting that TMEM99‐FUBP3‐p21 is a pathway.

p21 is one of the most classic cell cycle proteins, known for its roles in regulating the cell cycle and autophagy. In the past reports, it was mainly identified as a tumor suppressor, being a downstream protein of p53. Interestingly, in contrast to its anti‐proliferative activity, p21 has also been proven to promote cell survival through various mechanisms, including inhibiting cell apoptosis, stimulating cell motility, and supporting the assembly of cyclin D‐CDK4/6 complexes.^[^ ^36^ ^]^ Therefore, the role of p21 in maintaining cell homeostasis is more complicated than initially predicted, demonstrating functional “antagonistic duality”, especially in regulating cell death mechanisms.^[^ ^37^ ^]^ For this reason, we further verified and found that p21 mainly interacts directly with KH1 and KH4 of FUBP3 through its 3′UTR sequence. We also found that after knocking down TMEM99, FUBP3 overexpression could partially restore part of the expression of p21, but not to the initial level, which is illogical. Regarding this, we believe that control of p21 by TMEM99 through FUBP3 is not the only way; there might be another key factor involved.

In the RNA pull‐down and mass spectrometry analysis, IGF2BP2 was identified, which binds RNA through its six characteristic RNA‐binding domains, including two RNA Recognition Motifs (RRM1 and RRM2) and four KH domains (KH1‐KH4).^[^ ^38^ ^]^ IGF2BP2, a member of the m6A methylation reader protein family IGF2BPs, plays a crucial role in the development of cancer. It was initially identified as an m6A reader in cancer in 2019. Li et al.^[^ ^39^ ^]^ found that IGF2BP2 promotes SOX2 expression in colorectal cancer cells through an m6a‐IGF2BP2‐dependent mechanism, thereby promoting the development of colorectal cancer. In recent years, more studies have focused on IGF2BP2, proving its increasingly significant role in cancer.^[^ ^40^, ^41^ ^]^ Past reports indicate its ability to regulate p21 mRNA.

We found that IGF2BP2 can bind to TMEM99. Although past reports have shown that in lung adenocarcinoma, IGF2BP2 can affect cell proliferation, migration, and invasion, we found that knocking down TMEM99 does not affect IGF2BP2 expression and vice versa. However, when IGF2BP2 is knocked down, the abundance of p21 can be significantly reduced. Furthermore, we discovered that TMEM99 primarily binds to IGF2BP2 through its N‐terminal. As we previously mentioned, p21 has often been implicated as an oncogene affecting the cell death process in past studies, most commonly through cellular autophagy. Therefore, further investigations using electron microscopy and western blot analysis revealed that downstream p21 primarily promotes tumor development and progression by inhibiting autophagy.

We sought to understand how TMEM99, FUBP3, and IGF2BP2 collectively regulate p21. Our assays with Actinomycin D revealed that both FUBP3 and IGF2BP2 are required to stabilize p21 mRNA. Further coIP assays revealed no direct interaction between FUBP3 and IGF2BP2. Since IGF2BP2 is an m6A recognition protein, potential m6A sites in TMEM99 and p21 mRNA were predicted using the SRAMP database and confirmed by m6A RIP in the respective regions. Mutational analysis verified that IGF2BP2 binds to TMEM99 and p21 via m6A methylation. After knocking down FUBP3, the binding ability of IGF2BP2 with p21 significantly decreased. Interestingly, while FUBP3's ability to protect p21 mRNA stability decreases when IGF2BP2 is knocked down, RIP showed that FUBP3's binding ability with p21 is not affected by IGF2BP2 expression or p21 m6A sites. Notably, in the p21‐wt groups, overexpression of FUBP3 and IGF2BP2 led to a modest increase in m6A‐modified p21. However, no changes were observed in the p21‐mut groups. We hypothesize that the lack of significant upregulation in the p21‐wt groups may be due to the limited expression of TMEM99, which acts as a bridge.

We observed that, unlike previous reports where the binding of IGF2BP2 with lncRNA directly affected its expression or function, in this context, TMEM99 seems to alter the spatial conformation of IGF2BP2 after binding, enhancing its binding ability with p21. It also serves as a bridge, binding p21 through FUBP3, recruiting IGF2BP2, and reducing the spatial distance between IGF2BP2 and p21, thereby promoting p21 expression.

On the other hand, lncRNAs can initiate translation through Internal Ribosome Entry Site (IRES) or N6‐methyladenosine (m6A).^[^ ^42^, ^43^, ^44^ ^]^ We found that TMEM99 is indeed m6A modified. Thus, besides acting as a bridge for IGF2BP2 and p21 interaction, we conducted additional assays to determine whether TMEM99 might encode peptides. Through polysome profiling assays, we observed that from the position of monoribosomes (80s) (region 5), TMEM99 expression is almost zero, being entirely concentrated in the free RNA (regions 1–4), indicating no ribosome binding ability (Figure S8A–D, Supporting Information). We further employed the ORF finder to predict potential open reading frames (ORFs) and found that only four possible ORFs within TMEM99 were encoded, of which two have more than 40 amino acids (aa) (Figure S8E, Supporting Information). Construction of flag plasmids to inspect translation further validated that TMEM99 does not have the ability to encode peptides (Figure S8F, Supporting Information).

## 4 Conclusion

In summary, we have identified lncRNA TMEM99 as an oncogene in lung adenocarcinoma, closely associated with poor patient prognosis due to its role in cellular apoptosis and autophagy modulation. Mechanistically, TMEM99 binds via its tail end to the KH1 and KH3 domains of FUBP3, enhancing its stability. This interaction allows FUBP3 to attach to the 3′ UTR of p21 mRNA through its KH1 and KH4 domains. The TMEM99‐FUBP3 complex recruits IGF2BP2, promoting its binding to m6A sites within TMEM99 and p21, which stabilizes p21 mRNA and increases p21 protein levels. While this elevation in p21 typically inhibits autophagy, promoting tumor progression, our latest findings reveal that knocking down TMEM99 reverses this effect, leading to enhanced autophagy and impacting cellular apoptosis significantly.

## 5 Experimental Section

### RNA Sequencing and Calculation of Gene Expression

The RNAseq data presented in this study were from the previously published studies (24; 25). Briefly, RNA was extracted from the tumor and matched normal tissues of 197 lung adenocarcinoma patients and then sent for sequencing. Of them, 150 samples with RNA integrity number (RIN) ≥ 5.0 were included in the final analysis, including 16 adenocarcinomas in situ (AIS), 52 minimally invasive adenocarcinomas (MIA), and 82 invasive adenocarcinomas (LUAD). RNAseq reads were aligned against the reference human genome (hg19) using STAR v2.5.3.^[45]^ Raw reads were then normalized to the transcripts per million (TPM) estimates for downstream analyses using RSEM v1.3.0.^[46]^

### LUAD Tissue Specimen

A total of 27 patients who underwent surgery at the Fudan University Shanghai Cancer Center from 2020 to 2021 were selected for the present study. The specimen was stored in liquid nitrogen immediately after excision. All LUAD tissue‐related assays were approved by the Ethical Committee of the Fudan University Shanghai Cancer Center according to the Declaration of Helsinki. All patients signed informed consent prior to enrollment.

### Establishment of Stable Knockdown and Overexpressed LUAD Cell Lines

PLKO.1 plasmid was selected for shRNA construction, while the PCDH plasmid was used for overexpression. The packaging system uses the VSVG‐PMDL‐REV system. 293T cell lines were co‐transfected with virus vectors and target gene plasmids. After incubation for 48–72 h, the supernatant was collected and filtered to infect LUAD cell lines. For shRNA sequence information, see Table S1 (Supporting Information).

### Differential Expression Analysis

Analysis of differential expression genes between tumor and normal tissues was performed using the *R* package DESeq2 (47). Differentially expressed genes were defined as those genes with a fold‐change of no less than 1.5 times more highly expressed in either group. The *p*‐value < 0.01 was defined as significant. RNAseq data from the TCGA‐LUAD cohort were subsequently used to validate the findings. Significantly differentially expressed lncRNAs were listed according to their p‐values. Volcano plots were generated using the *R* package EnhancedVolcano.

### Cell Lines

LUAD cell lines (H1299, A549, PC9, H1437, 5889, H23, H1975, and H358), the human bronchial epithelial cell line (Beas‐2B), and the 293T cell line were procured from the American Type Culture Collection (ATCC). Human cell lines were cultured in DMEM medium, supplemented with 10% Fetal Bovine Serum and 1% penicillin‐streptomycin, and maintained at 37 °C in a 5% CO2 atmosphere. All cell lines tested negative for Mycoplasma.

### Xenograft Mouse Model

All animal assays adhered strictly to the principles and procedures sanctioned by the Ethical Committee of Animal Experiments at the Fudan University Shanghai Cancer Center. H1299 cells (5×10^6) were injected into each 6‐week‐old female mouse, with subsequent tumor measurements performed every five days. Once the tumor reached a volume of 200 mm^3, mice were systematically divided into four groups (*n* = 5): control, paclitaxel (PTX) (Selleck), antisense oligonucleotides (ASO) (RiboBio), and a combination of paclitaxel and ASO, with liquid dosages in each group compensated by the corresponding treatment group's solvent. ASO reagent was administered intratumorally. To prevent ASO liquid spillage, initial injections were 2 nmol (100 mM ASO, 20 µL) per mouse, followed by 5 nmol (100 mM ASO, 50 µL) every five days. Paclitaxel (10 mg kg^−1^, 200 µL Cremophor EL) was intraperitoneally injected every five days. Tumor dimensions were recorded every five days and volume calculated using the formula: Volume = (length × width^2)/2

### Transfection

Cells were transfected with siRNA sequences and target plasmids using Lipofectamine 2000 reagent (Thermo Fisher), in accordance with the manufacturer's instructions. The cells were then utilized for subsequent assays 48–72 h post‐transfection.

### Real‐Time qRT‐PCR

Total RNA was extracted from cells utilizing Trizol, per the operational manual. Subsequently, cDNA was synthesized utilizing the Superscript First‐Strand cDNA Synthesis Kit (TAKARA), and qRT‐PCR was performed employing Power SYBR Green PCR master mix (Yeasen Biotechnology) on a QuanStudioTM 7 Flex Real‐Time PCR System (Thermo Fisher). Primer details are provided in Table S2 (Supporting Information).

### Cell Proliferation Assay

Cell proliferation was assessed using the Cell Counting Kit‐8 (CCK8) reagent (Dojindo). Cells, either transfected with siRNA or established from stable cell lines, were seeded at 2000 cells per well in 96‐well plates. Following cell adherence, the medium was replaced with a complete culture medium containing 10% CCK8 and incubated at 37 °C and 5% CO2 for 2 h. Absorbance was read spectrophotometrically at 450 nm, and raw values were utilized directly for plotting. Each experimental condition was replicated in five duplicate wells.

### Transwell Migration and Invasion Assays

Transwell chambers (Magna) were utilized for both migration and invasion assays. For migration, 4 × 10^4^ cells were resuspended in 200 µL serum‐free DMEM and seeded into the upper chamber, while 600 µL of complete medium was added to the wells of a 24‐well plate. For invasion assays, the cell number was doubled, and the chamber was pre‐coated with Matrigel (Magna). Post‐24 h incubation, chambers were fixed in 4% paraformaldehyde (30 min) and stained with 0.1% crystal violet (30 min). Residual cells and Matrigel inside the chamber were removed, followed by cell imaging and counting using a DP2‐BSW 2.2 (OLYMPUS).

### Western Blot Analysis

Cells were washed with cold PBS and lysed using RIPA buffer supplemented with a protease and phosphatase inhibitor cocktail (CST) and PMSF. Following DNA shearing via ultrasonication and a 30 min ice incubation, insoluble impurities were eliminated by centrifugation. Protein quantification employed the BCA Protein Assay Kit (Thermo Fisher). Equal protein amounts were resolved by SDS‐PAGE and transferred to PVDF membranes (Bio‐Rad). Membranes were incubated with primary antibodies (Table S3, Supplementary Table) overnight at 4 °C. After secondary antibody incubation, blots: RIP assays were conducted using the RNA‐Binding Protein Immunoprecipitation Kit (Magna), following the manufacturer's instructions. Post‐PBS wash, cell lysates were incubated overnight at 4 °C with anti‐FUBP3, anti‐IGF2BP2, and IgG antibodies. Magnetic beads were added and incubated for 1 h at room temperature, followed by three gentle washes. Finally, RNA was eluted for qRT‐PCR quantification.

### RNA Pull‐Down Assay

Target bands were transcribed in vitro and biotin‐labeled using the Pierce RNA 3′ End Biotinylation Kit (Thermo Fisher). Post‐cold PBS wash, cells were lysed with RIPA buffer containing a protease inhibitor. The RNA pull‐down assay was conducted using a kit (Thermo Fisher), strictly adhering to the provided instructions, and concluded with protein solution elution. Equal protein amounts were resolved via SDS‐PAGE and silver‐stained using a kit (Beyotime) to visualize differential proteins.

### Fluorescence In Situ Hybridization (FISH)

Cells were fixed in paraformaldehyde (10 min), treated with 20% acetic acid, dehydrated with ethanol, and pre‐hybridized at 60 °C (3% BSA, 4 × SSC) for 1 h. Hybridization employed a buffer containing probe (10% DSS, 4 × SSC). For simultaneous RNA and protein localization, post‐probe hybridization solution wash and a 5% BSA blocking step (1 h), primary and secondary antibodies were incubated at room temperature for 1 h, with intermediary PBST washes. Subsequent DAPI incubation (10 min, room temperature) was followed by imaging using a CarlZeiss LSM710 confocal microscope (Germany).

### Immunohistochemistry

Adhering to standard immunohistochemical procedures, paraffin sections were incubated at 65 °C for 1 h, followed by dewaxing via xylene and ethanol, and permeabilization with 0.5% Triton X‐100 for 20 min. Endogenous peroxidase was quenched with 3% H2O2 for 10 min, slides were blocked using goat serum for 30 min, and subsequently incubated with primary antibody overnight at 4 °C and with secondary antibody for 1 h at 37 °C. Slides were stained with DAB, counterstained with hematoxylin, and finalized with an ammonia reversion step. Antigen levels were visualized and analyzed using cellSens Standard 1.18 (OLYMPUS).

### Co‐Immunoprecipitation (CoIP)

Magnetic beads and respective antibodies, with IgG as a control, were combined with binding buffer (Novagen) and incubated at 4 °C for 2 h. Cells, grown to 90% confluence in a 10 cm dish, were washed with cold PBS and lysed using RIPA. After ultrasonication, equal parts of the cell lysate mix were added to each antibody‐bead group and incubated overnight at 4 °C. Magnetic beads were collected, washed thrice with binding buffer, and eluted proteins were verified via western blot.

### Colony Formation Assay

A 2000‐cell culture from the stably transformed cell line was seeded into 6‐well plates and cultured for 15 days. Cells were washed twice with cold PBS, fixed with 4% paraformaldehyde at 4 °C for 10 min, washed again, and stained with 0.1% crystal violet for 30 min.

### MG132, CHX, and Actinomycin D Assays

Cells were seeded in a 6‐well plate, maintaining a density between 50%–70%. Subsequently, cells were treated with complete medium containing MG132 (5 µM), cyclohexane (CHX) (20 µg mL^−1^), or actinomycin D (2 mg mL^−1^) at designated time points (0, 40, 80, and 120 min). For MG132 and CHX, incubation durations were 120, 80, 40, and 0 min, respectively, and protein expression alterations were analyzed via western blotting. For actinomycin D, incubation was set at 0, 12, and 24 h, with affected mRNA evaluated through qRT‐PCR.

### m6A‐RIP (m6A RNA Immunoprecipitation)

Adhering to the standardized procedure outlined by the m6A Transcriptome Profiling Kit (RiboBio), total RNA was fragmented by incorporation of RNA Fragmentation Buffer. Subsequently, magnetic beads A/G and the m6A antibody were incubated together at room temperature for 30 min. Fragmented RNA and magnetic beads A/G were amalgamated and gently rotated in the MeRIP reaction solution for 2 h at 4 °C. After RNA elution from magnetic beads, RNA was extracted, then validated via reverse transcription and qRT‐PCR analysis.

### Polysome Profiling

Cell lysates, containing RNAse inhibitors and ribosome fixing solution, were layered onto 10%–50% sucrose gradients and ultracentrifuged at 39 000 rpm for 2 h at 4 °C. Following ultracentrifugation, fractions were meticulously collected from top to bottom into separate tubes for RNA extraction. Ribosomal absorbance was monitored throughout the fractionation process. After the RNA extraction, the RNA content within each fraction was analyzed using quantitative PCR (qRT‐PCR).

### Study Approval

Clinical LUAD tissues were obtained from patients who provided written informed consent prior to their inclusion in the study. This study was approved by the Ethics Committee of Sun Fudan University Shanghai Cancer Center (approval No: 050432‐4‐1911D). All animal experiments were conducted in accordance with the guidelines of the Animal Care and Use Committee of Fudan University Shanghai Cancer Center (approval No: FUSCC‐IACUC‐S20210232).

### Statistical Analysis

Graphs from transwell and FISH assays were processed using ImageJ software (v1.52a, USA). Statistical analyses were conducted with R (v4.2.1), GraphPad Prism (v9.0), and SPSS (v26.0). All experiments were independently repeated at least three times (N ≥ 3), and data are presented as mean ± SEM. Two‐group comparisons were evaluated using Student's t‐test, while one‐way ANOVA followed by Dunnett's post hoc test was applied for multiple group comparisons. Pearson correlation analysis was used to assess linear relationships. A *p*‐value > 0.05 was considered not significant (n.s.); significance was indicated as follows: *p* < 0.05; *p* < 0.01; *p < 0.001.

### Ethics Approval and Consent to Participate

This research was approved by the Ethics Committee of FUSCC and Fudan University. All samples were obtained with informed consent.

### Consent for Publication

All subjects have written informed consent.

## Conflict of Interest

The authors declare no conflict of interest.

## Author Contributions

Z.W. and Y.Z. contributed equally to this work. Y.S., Z.W., Y.Z., and Y.P. designed and performed the research. Z.W., P.L., Y.W., Y.S., and X.C. collected data, and Y.Z., Q.H., D.D.Z., and C.Y. performed statistical analysis. Z.W. and H.H. wrote the draft manuscript. All authors contributed to the writing and reviewing of the manuscript and approved the final manuscript for submission.

## Supporting information



Supporting Information

Supporting Information

Supplementary Table

## Data Availability

Source data for figures and Additional file three have been provided as Statistics Source Data. The RNA‐seq data have been submitted to the Gene Expression Omnibus (https://ngdc.cncb.ac.cn/gsa‐human/), and the data can be accessed by the accession number HRA010942. Other data supporting the findings of this study are available from the corresponding authors.
