# Correction to “LncRNA TMEM99 Complexes with IGF2BP2 to Inhibit Autophagy in Lung Adenocarcinoma”

**DOI:** 10.1002/advs.202521888

**Published:** 2025-11-27

Wu Z, Zhao Y, Peng Y, Liu P, Huang Q, Wo Y, Pan Y, Zheng D, Yuan C, Shang Y, Chen X, Hong H, Sun Y. LncRNA TMEM99 Complexes with IGF2BP2 to Inhibit Autophagy in Lung Adenocarcinoma. Adv Sci (Weinh). 2025 Sep;12(33):e07871. doi: 10.1002/advs.202507871.


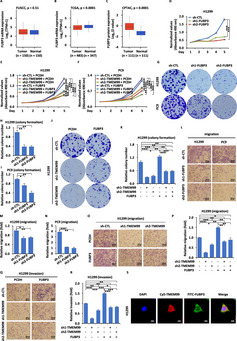


In Figure 4Q, the image for the “sh2‐TMEM99” group under the FUBP3 overexpression condition is incorrect. The correct image is the one I have resubmitted and should be used as the replacement.

We wish to emphasize that this error is limited to the presentation of this specific panel and does not affect the main conclusions or novelty of the study. Consequently, no modifications to the text of the article are required.

This oversight appears to have occurred during figure assembly, likely due to an accidental drag‐and‐drop error while arranging the panels. Although image‐duplication checks were performed prior to the initial submission, this mistake was unfortunately introduced during the final proofing stage.

We sincerely apologize for this oversight.

