# Supplementary material for: LncRNA TMEM99 Complexes with IGF2BP2 to Inhibit Autophagy in Lung Adenocarcinoma
Source: Adv Sci (Weinh). 2025 Jul 24;12(33):e07871. doi: 10.1002/advs.202507871 (PMC12412623; doi:10.1002/advs.202507871)
Supplement: Supplementary file 1 — Supporting Information [file ADVS-12-e07871-s003.pdf]

ADVANCED SCIENCE
Open Access

Supporting Information

for *Adv. Sci.*, DOI 10.1002/advs.202507871

LncRNA TMEM99 Complexes with IGF2BP2 to Inhibit Autophagy in Lung Adenocarcinoma

Zhigang Wu, Yue Zhao, Yizhou Peng, Pengcheng Liu, Qixuan Huang, Yang Wo, Yunjian Pan, DongDong Zheng, Chongze Yuan, Yan Shang, Xiao Chen, Hui Hong and Yihua Sun**

Supplementary Figure

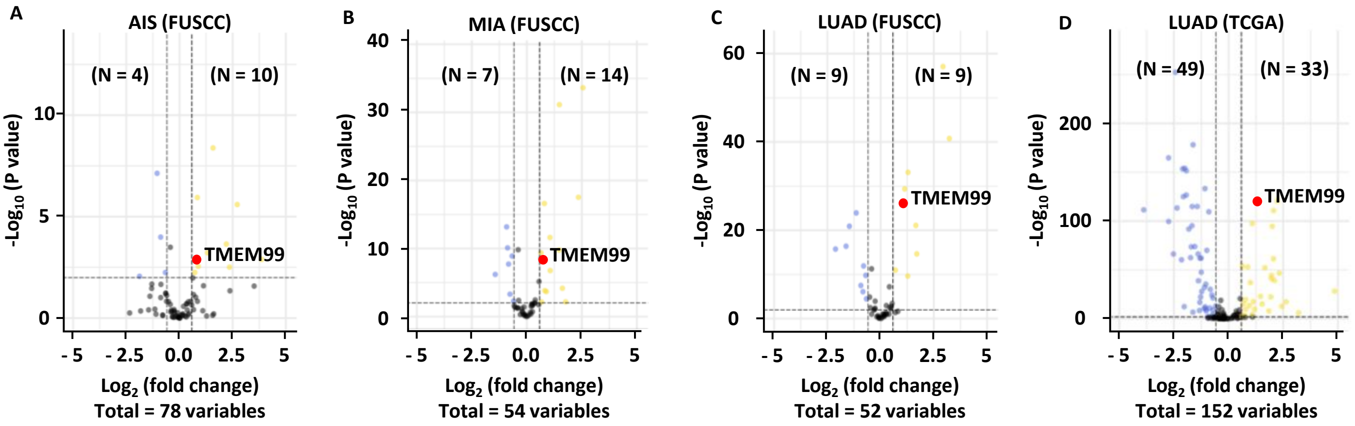

Figure S1

Supplementary Figure

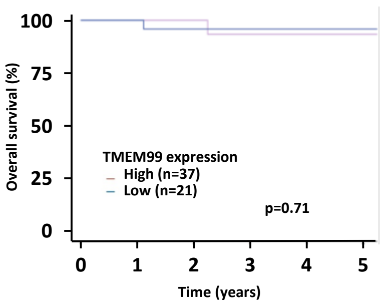

Figure S2

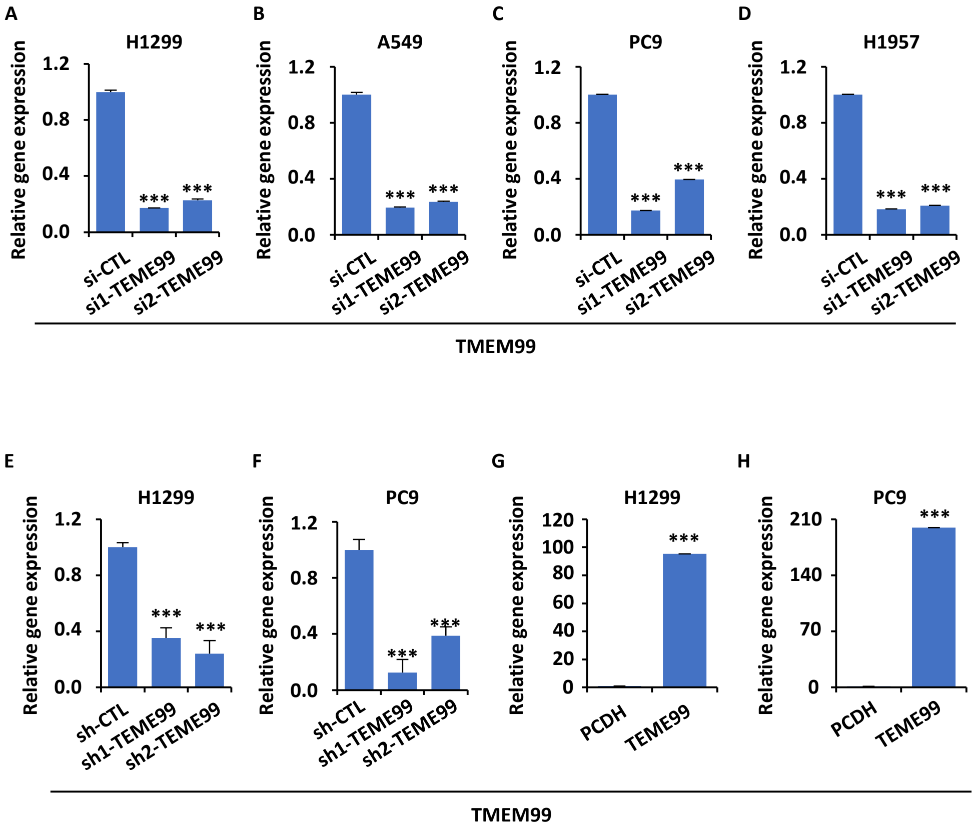

A
H1299
Relative gene expression
1.2
0.8
0.4
0.0
***
***
si-CTL
si1-TEME99
si2-TEME99
B
A549
C
PC9
D
H1957
TMEM99
E
H1299
sh-CTL
sh1-TEME99
sh2-TEME99
F
PC9
G
H1299
120
100
80
60
40
20
0
PCDH
TEME99
H
PC9
210
140
70
0
TMEM99

Figure S3

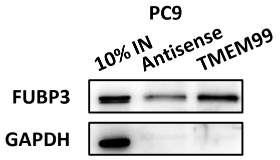

Figure S4

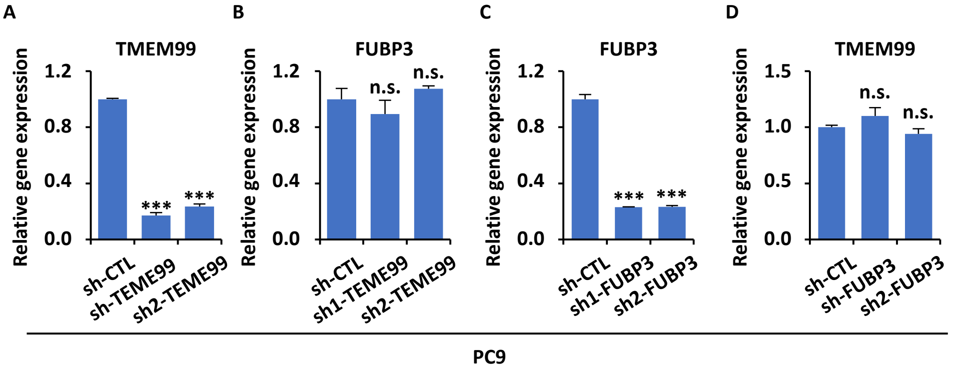
A
TMEM99
Relative gene expression
1.2
0.8
0.4
0.0
***
***
sh-CTL
sh-TEME99
sh2-TEME99
B
FUBP3
Relative gene expression
1.2
0.8
0.4
0.0
n.s.
n.s.
sh-CTL
sh1-TEME99
sh2-TEME99
C
FUBP3
Relative gene expression
1.2
0.8
0.4
0.0
***
***
sh-CTL
sh1-FUBP3
sh2-FUBP3
D
TMEM99
Relative gene expression
1.5
1.0
0.5
0.0
n.s.
n.s.
sh-CTL
sh-FUBP3
sh2-FUBP3
PC9

Figure S5

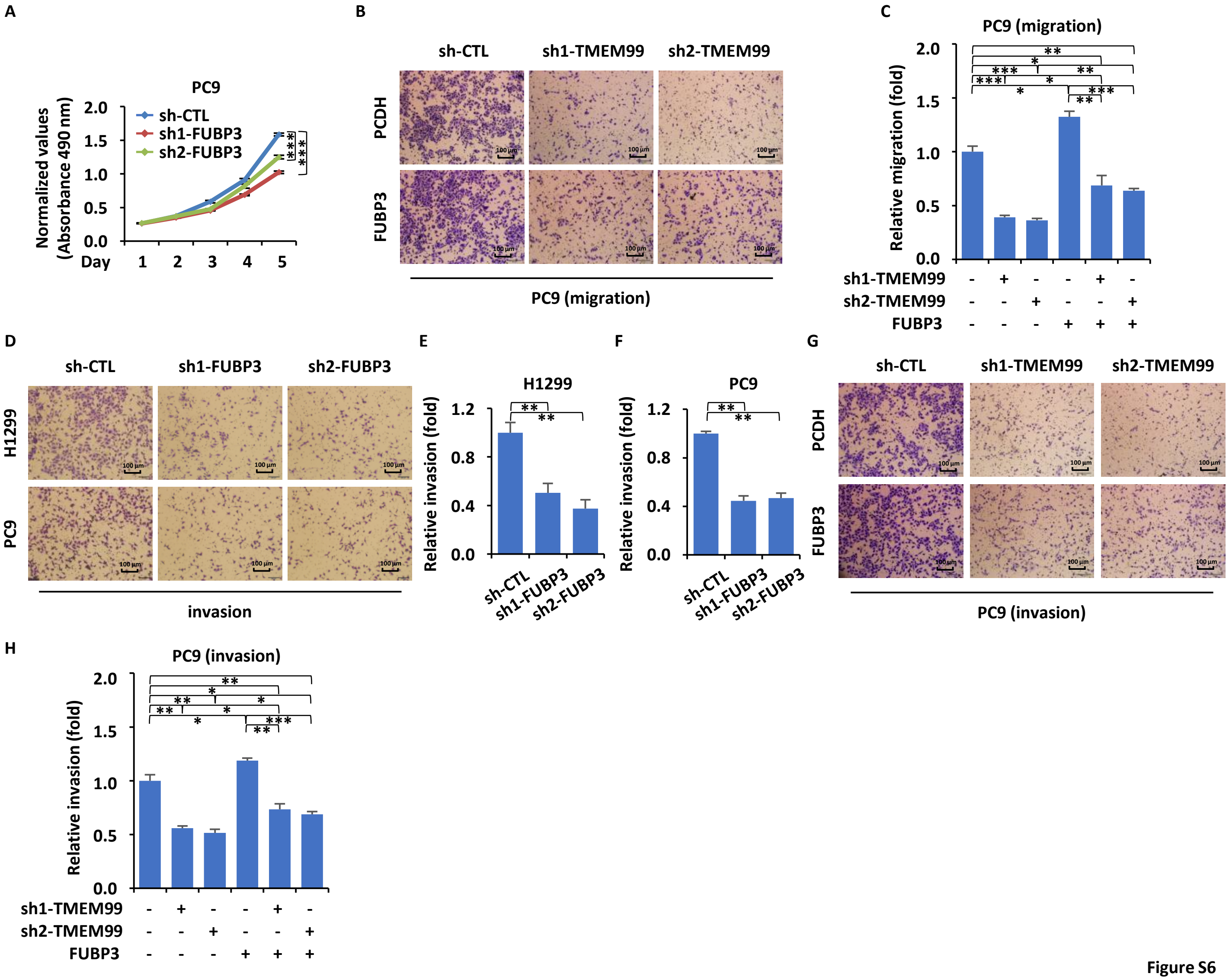
A
PC9
Normalized values (Absorbance 490 nm)
sh-CTL
sh1-FUBP3
sh2-FUBP3
Day
B
sh-CTL
sh1-TMEM99
sh2-TMEM99
PCDH
FUBP3
PC9 (migration)
C
PC9 (migration)
Relative migration (fold)
sh1-TMEM99
sh2-TMEM99
FUBP3
D
sh-CTL
sh1-FUBP3
sh2-FUBP3
H1299
PC9
invasion
E
H1299
Relative invasion (fold)
F
PC9
Relative invasion (fold)
G
sh-CTL
sh1-TMEM99
sh2-TMEM99
PCDH
FUBP3
PC9 (invasion)
H
PC9 (invasion)
Relative invasion (fold)
sh1-TMEM99
sh2-TMEM99
FUBP3

Figure S6

A

B

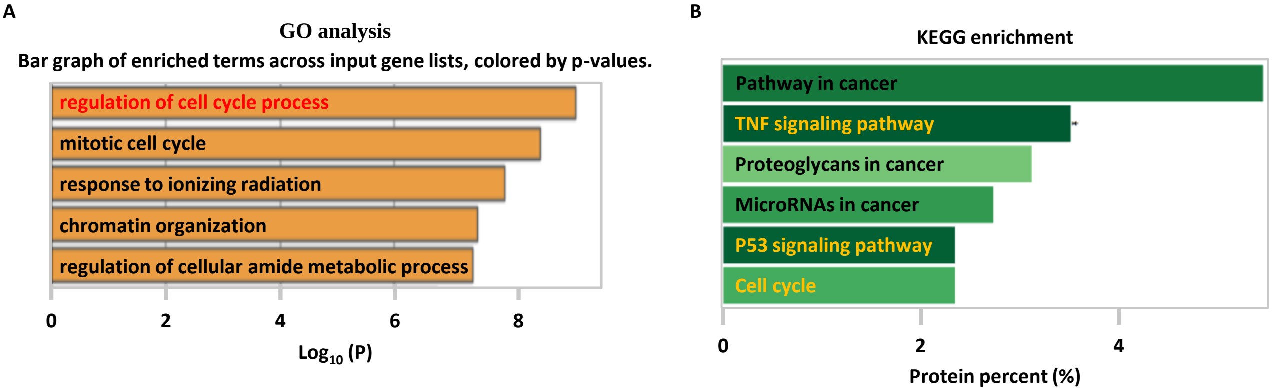
GO analysis
Bar graph of enriched terms across input gene lists, colored by p-values.
regulation of cell cycle process
mitotic cell cycle
response to ionizing radiation
chromatin organization
regulation of cellular amide metabolic process
0
2
4
6
8
Log10 (P)
KEGG enrichment
Pathway in cancer
TNF signaling pathway
Proteoglycans in cancer
MicroRNAs in cancer
P53 signaling pathway
Cell cycle
0
2
4
Protein percent (%)

Figure S7

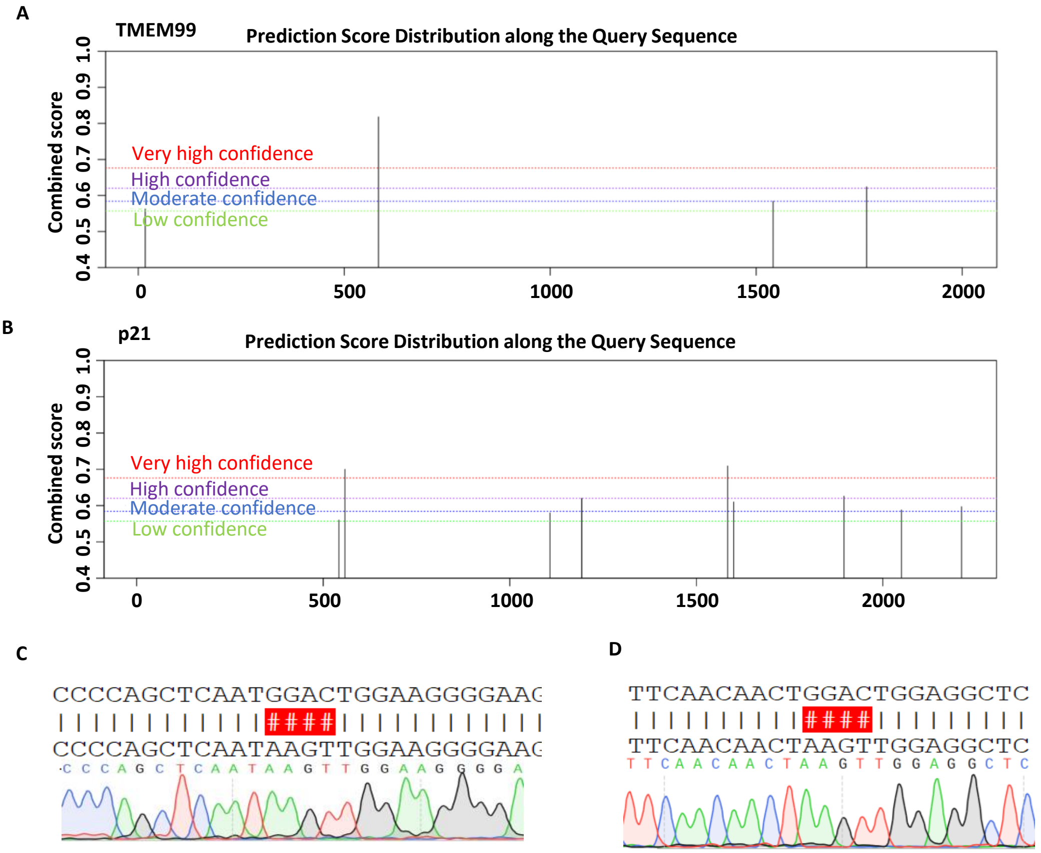
A
TMEM99
Prediction Score Distribution along the Query Sequence
Combined score
0.4 0.5 0.6 0.7 0.8 0.9 1.0
Very high confidence
High confidence
Moderate confidence
Low confidence
0
500
1000
1500
2000
B
p21
Prediction Score Distribution along the Query Sequence
Combined score
0.4 0.5 0.6 0.7 0.8 0.9 1.0
Very high confidence
High confidence
Moderate confidence
Low confidence
0
500
1000
1500
2000
C
CCCCAGCTCAATGGACTGGAAGGGGAAG
####
CCCCAGCTCAATAAGTTGGAAGGGGAAG
D
TTCAACAACTGGACTGGAGGCTC
####
TTCAACAACTAAGTTGGAGGCTC
T T C A A C A A C T A A G T T G G A G G C T C

Figure S8

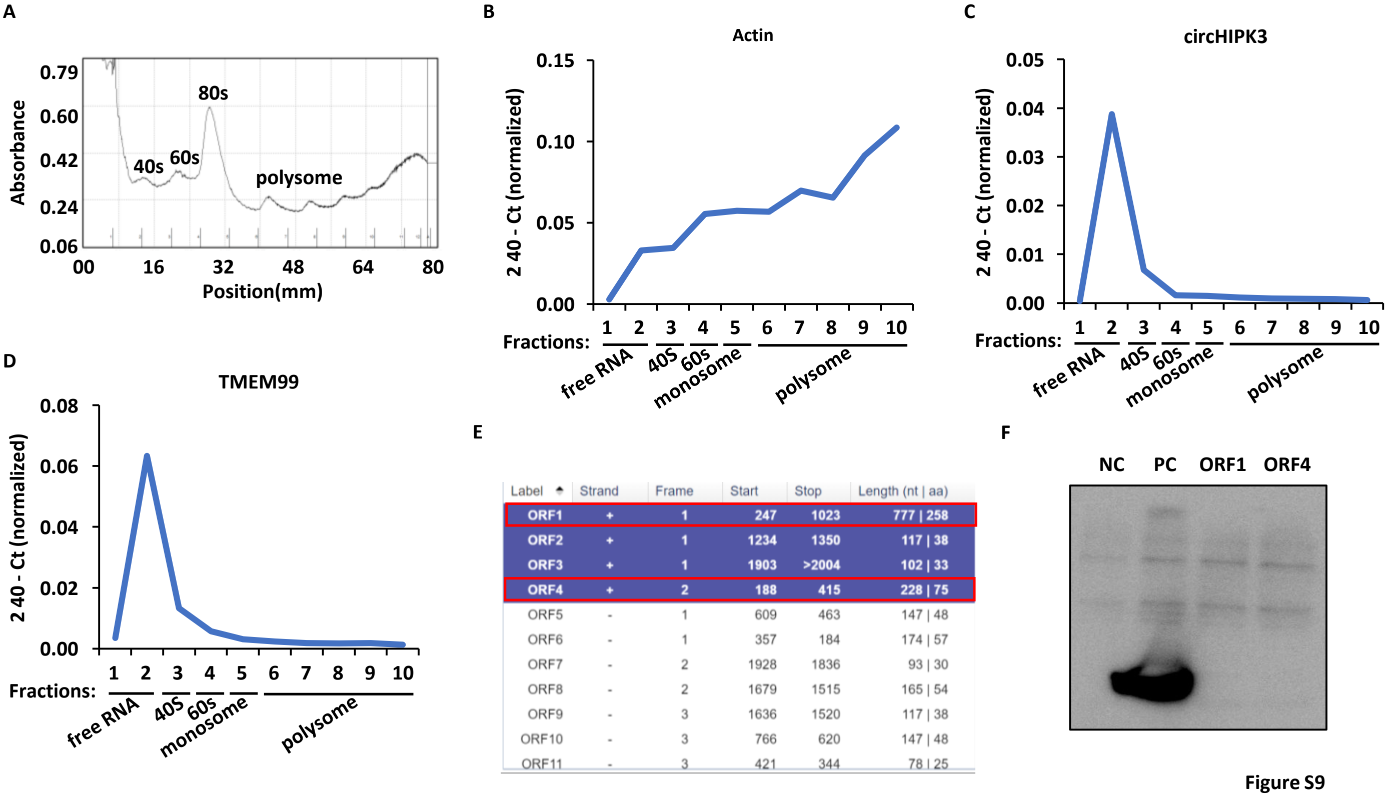

Label	Strand	Frame	Start	Stop	Length (nt | aa)
ORF1	+	1	247	1023	777 | 258
ORF2	+	1	1234	1350	117 | 38
ORF3	+	1	1903	>2004	102 | 33
ORF4	+	2	188	415	228 | 75
ORF5	-	1	609	463	147 | 48
ORF6	-	1	357	184	174 | 57
ORF7	-	2	1928	1836	93 | 30
ORF8	-	2	1679	1515	165 | 54
ORF9	-	3	1636	1520	117 | 38
ORF10	-	3	766	620	147 | 48
ORF11	-	3	421	344	78 | 25

Figure S9
