# Supplementary material for: LncRNA TMEM99 Complexes with IGF2BP2 to Inhibit Autophagy in Lung Adenocarcinoma
Source: Adv Sci (Weinh). 2025 Jul 24;12(33):e07871. doi: 10.1002/advs.202507871 (PMC12412623; doi:10.1002/advs.202507871)
Supplement: Supplementary file 2 — Supporting Information [file ADVS-12-e07871-s001.docx]

**Supplementary Figure Legend
Figure S1.** **Distribution of high expression of lncRNA in lung adenocarcinoma. Related to Figure 1.** A-D) Volcano plot analysis of lncRNAs in AIS, MIA, LUAD using FUSCC and TCGA data (FC > 1.5, P < 0.01).

**Figure S2.** **LncRNA TMEM99 is an important oncogene in the prognosis of lung adenocarcinoma. Related to Figure 1.** The influence of TMEM99 on overall survival (OS) in lung adenocarcinoma based on data from FUSCC (P = 0.71). Statistical significance was determined by the log-rank test.

**Figure S3. TMEM99 expression levels and knockdown and overexpression efficiency in various lung** **adenocarcinoma cell lines. Related to Figure 1.** A-D) H1299 (A), A549 (B), PC9 (C), and H1975 (D) cell lines transfected with si-CTL, si1-TMEM99 or si2-TMEM99 were subjected to qRT-PCR analysis of expression level of TMEM99 (± SEM; ***P < 0.001). E-F) H1299 (E, G) and PC9 (F, H) cells infected with sh-CTL, sh1/2-TMEM99, PCDH and TMEM99-vector followed by qRT-PCR to examine TMEM99 expression. The data are shown as the mean ± SD (n = 3). ***p < 0.001, by two-tailed unpaired Student’s t test (G, H) and one-way ANOVA followed by Dunnett’s post hoc test (A-F).

**Figure S4. TMEM99 interacts with FUBP3 in PC9 cells. Related to Figure 3.** *In vitro* TMEM99 RNA-pull down assay was performed, and the protein levels of FUBP3 in PC9 was analyzed by IB analysis in PC9.

**Figure S5. Interplay between TMEM99 and FUBP3 in PC9 cells.** **Related to Figure 3.** A, B) PC9 cells infected with sh-CTL, sh1/2-TMEM99 followed by qRT-PCR to examine TMEM99 (A) and FUBP3 mRNA (B) expression (± SEM; ***P < 0.001; n.s. not significant). C, D) PC9 cells infected with sh-CTL, sh1/2-FUBP3 followed by qRT-PCR to examine FUBP3 (C) and TMEM99 (D) expression (± SEM; ***P < 0.001; n.s. not significant). The data are shown as the mean ± SD (n = 3) by one-way ANOVA followed by Dunnett’s post hoc test (A-D).

**Figure S6. Functional characterization of FUBP3 in PC9 cell lines.** **Related to Figure 4.** A) PC9 cells were infected with sh-CTL, sh1/2-FUBP3 were subjected to proliferation assay (± SEM; ***P < 0.001). B) PC9 cells were infected with sh-CTL, sh1/2-TMEM99 were transfected with FUBP3 or not as indicated followed by proliferation assay. C) Quantification of cell numbers as shown in (B) (± SEM; *P < 0.05; **P < 0.01; ***P < 0.001). D) H1299 or PC9 cells were infected with sh-CTL, sh1/2-FUBP3 were subjected to invasion assay. E, F) Quantification of H1299 (E) or PC9 (F) cell numbers as shown in (D) (± SEM; *P < 0.05; **P < 0.01; ***P < 0.001). G) PC9 cells described in (B) were subjected to invasion assay. H) Quantification of cell numbers as shown in (G) (± SEM; *P < 0.05; **P < 0.01; ***P < 0.001). The data are shown as the mean ± SD (n = 3) by one-way ANOVA followed by Dunnett’s post hoc test (A, C, E, F, H).

**Figure S7. RNAseq analysis and validation of downstream genes altered by FUBP3 modulation. Related to Figure 5.** A) Gene Ontology analysis (GO analysis) of the five most enriched processes for gene positively regulated by FUBP3 in H1299 cells. B) KEGG pathway analysis of the six most enriched pathways for gene positively regulated by FUBP3 in H1299 cells.

**Figure S8. Prediction of m6A sites in genes and construction of stable clones with site mutations. Related to Figure 7.** A, B) Identification of m6A methylation sites in TMEM99 (A) and p21 (B) mRNA using the SRAMP database. C, D) Stable plasmids were constructed and Sanger sequencing was performed to detect m6A site mutations in TMEM99 (C) and p21 mRNA (D).

**Figure S9. Investigation into the peptide-coding potential of TMEM99. Related to Figure 7.** A) Polysome profiling assessing ribosomal distribution in H1299; B-D) H1299 cells were subjected to polysome profiling assay, and the resultant fractions were subjected to RNA extraction and qRT-PCR analysis to examine the expression of Actin (B), circHIPK3 (C) or TMEM99 (D). Fraction 1-2: free RNA (unbound with ribosome); Fraction 3: 40S (40S ribosomal subunit); Fraction 4: 60S (60S ribosomal subunit); Fraction 5: monosome; Fraction 6-10: polysome. E) ORF finder predicting potential gene ORFs encoding short peptides; F) IB analysis of gene ORF-Flag plasmid expression in H1299 cells.
