# Supplementary material for: LncRNA TMEM99 Complexes with IGF2BP2 to Inhibit Autophagy in Lung Adenocarcinoma
Source: Adv Sci (Weinh). 2025 Jul 24;12(33):e07871. doi: 10.1002/advs.202507871 (PMC12412623; doi:10.1002/advs.202507871)
Supplement: Supplementary file 3 — Supplementary Table [file ADVS-12-e07871-s002.docx]

**Supplementary tables**

Table 1. List of shRNA sequences

| Plko1 | shRNA sequence |
| --- | --- |
| sh1-TMEM99 | F: CCGGCACCCATTATGGAGATTAACTC  GAGTTAATCTCCATAATGGGTGTTTTTG |
|  | R: AATTCAAAAACACCCATTATGGAGATT  AACTCGAGTTAATCTCCATAATGGGTG |
| sh2-TMEM99 | F: CCGGGGGAGAGAGTTGAAATGGTCTC  GAGACCATTTCAACTCTCTCCCTTTTTG |
|  | R: AATTCAAAAAGGGAGAGAGTTGAAAT  GGTCTCGAGACCATTTCAACTCTCTCCC |
| sh1-FUBP3 | F: CCGGGCAGCTCATAGATGAGAAACTC  GAGTTTCTCATCTATGAGCTGCTTTTTG |
|  | R: AATTCAAAAAGCAGCTCATAGATGAG  AAACTCGAG TTTCTCATCTATGAGCTGC |
| sh2-FUBP3 | F: CCGGGTGTGAGGATTCAGTTTAACTCG  AGTTAAACTGAATCCTCACACTTTTTG |
|  | R: AATTCAAAAAGTGTGAGGATTCAGTTT  AACTCGAG TTAAACTGAATCCTCACAC |
| sh1-IGF2BP2 | F: CCGGACCCACCAAACCAACCAATCACTC  GAGTGATTGGTTGGTTTGGTGGGTTTTTTG |
|  | R: AATTCAAAAAACCCACCAAACCAACCAAT  CACTCGAG TGATTGGTTGGTTTGGTGGGT |
| sh2-IGF2BP2 | F: CCGGAGCCATCAATATTCAGCAAAACTC  GAGTTTTGCTGAATATTGATGGCTTTTTTG |
|  | R: AATTCAAAAAAGCCATCAATATTCAGCAA  AACTCGAG TTTTGCTGAATATTGATGGCT |
| sh1- METTL3 | F: CCGGCAAGTATGTTCACTATGAACTCGAG  TTCATAGTGAACATACTTG TTTTTG |
|  | R: AATTCAAAAACAAGTATGTTCACTATGAA  CTCGAGTTCATAGTGAACATACTTG |
| sh2- METTL3 | F: CCGGGACTGCTCTTTCCTTAATACTCGAG  TATTAAGGAAAGAGCAGTC TTTTTG |
|  | R: AATTCAAAAAGACTGCTCTTTCCTTAATA  CTCGAGTATTAAGGAAAGAGCAGTC |

Table 2. List of primers.

| Gene | Primer sequence |
| --- | --- |
| β-Actin | F: CTCTTCCAGCCTTCCTTCCT |
|  | R: AGCACTGTGTTGGCGTACAG |
| MALAT1 | F: TGCTGTGTGCCAATGTTTCG |
|  | R: AATCCCCTAGGGAAGGGGTC |
| circHIPK3 | F: TCGGCCAGTCATGTATCAAA |
|  | R: CCCTTAGTGGGAGGATGAGA |
| TMEM99 | F: TTCTTCCTGGCTCTCCCTTG |
|  | R: CCCAATGGCCTCTGGTAAGA |
| FUBP3 | F: GCCTCTTCGTATCACTGGAGA |
|  | R: ACTGAATCCTCACACCAGCA |
| P21 | F: GACACCACTGGAGGGTGACT |
|  | R: GGCGTTTGGAGTGGTAGAAA |
| IGF2BP2 | F: AGAGAAGCCTGTCACCATCC |
|  | R: AGTGATGGTTCTTTCCGGGT |
| METTL14 | F: AGAAACTTGCAGGGCTTCCT |
|  | R: TCTTCTTCATATGGCAAATTTTCTT |
| TMEM99-FISH | TGATAGCCACCACCACCATATCTTGACAAG-CY5 |
| P21-FISH | ACCATGTGGACCTGTCACTGTCTTGTA-FITC |

Table 3. List of antibodies.

| Antibody | Brand | NO. |
| --- | --- | --- |
| PTBP1 | Abcam | Ab133734 |
| PTBP2 | Abcam | Ab154787 |
| PTBP3 | Novus | NBP1-80453 |
| FUBP3 | Abcam | Ab181025 |
| IGF2BP2 | Proteintech | 11601-1-AP |
| p21 | Abcam | Ab109520 |
| NOVA1 | Abcam | Ab183024 |
| GAPDH | Abcam | Ab9485 |
| LC3B | Abcam | Ab192890 |
| Atg5 | Abcam | Ab109490 |
| Atg7 | Abcam | Ab52472 |
| p62 | Abcam | Ab109012 |
| Actin | Abcam | Ab8226 |
| Beclin 1 | Abcam | Ab207612 |
| IgG (Rb) | Abcam | Ab205718 |
| IgG (Ms) | Abcam | Ab205724 |
